# Stress-Based Assessment of Bio-Inspired Phosphene Vision Encoding: Trade-Offs Among Performance, Residual Proxy Safety Burden, and Topology-Based Representation Metrics

**DOI:** 10.3390/biomimetics11070455

**Published:** 2026-07-01

**Authors:** Youngseok Lee

**Affiliations:** Department of Electronics, Chungwoon University, Incheon 22100, Republic of Korea; yslee@chungwoon.ac.kr

**Keywords:** phosphene vision, visual neuroprosthesis, simulated prosthetic vision, stress testing, multi-objective evaluation, residual proxy safety burden, topology-based analysis, operating-envelope characterization

## Abstract

Phosphene-based visual neuroprostheses require encoding schemes that preserve task-relevant information while remaining feasible under safety-constrained stimulation. This study proposes a stress-based evaluation framework that reframes phosphene encoder assessment as a tri-objective operating-envelope problem rather than a single-metric comparison. Four representative encoders—rate, sparse, temporal, and optim—were evaluated under structured perturbations using two simulated prosthetic-vision(SPV) benchmarks: EMNIST Letters for symbolic recognition and a COCO-derived balanced subset for a reduced four-class COCO-derived image-level classification task. The final experimental configuration used 20,000/3000/3000 train/validation/test samples for EMNIST and 4000/1000/1000 for the COCO-derived benchmark. The framework combined structured stress sweeps, residual proxy safety-burden analysis, topology-based representation metrics, and stress-integrated utility summaries within a simulation-based evaluation setting. The results showed that clean-conditioning accuracy alone was insufficient to predict encoder behavior under stress. EMNIST exhibited broad operator-dependent degradation, whereas the COCO-derived benchmark showed a lower but more compressed performance regime. Residual proxy safety burden was only loosely aligned with performance, with moderate dissociation between performance and residual proxy safety burden in EMNIST and weaker alignment between these two axes in the COCO-derived benchmark. In the point-estimate utility summaries, the sparse encoder tended to yield comparatively favorable tri-objective utility values within the present single-run simulation-based framework, simplified SPV percept-synthesis operator, and fixed benchmark-specific decoder setting, primarily because it maintained an almost negligible residual proxy safety burden while preserving competitive performance and topology-based representation metrics. Topology-based analysis further indicated that topology-based representation metrics largely tracked task degradation in EMNIST, whereas topology-based representation metrics showed larger relative variation than decoder accuracy within the evaluated simulation setting under degraded COCO-derived conditions. Taken together, these findings provide an exploratory, benchmark-specific assessment suggesting that phosphene encoder evaluation may benefit from a multi-axis operating-envelope-oriented analysis that jointly considers stressed functional performance, residual proxy safety burden, and topology-based representation metrics within the present simplified SPV and fixed-decoder evaluation setting. These results should therefore be interpreted as simulation-level, configuration-dependent observations under a simplified SPV percept-synthesis operator, with safety-related quantities treated as residual proxy safety-burden summaries rather than as direct physiological, electrochemical, clinical, or implant-specific safety measurements.

## 1. Introduction

Severe vision loss caused by late-stage retinal degenerations such as retinitis pigmentosa and advanced age-related macular degeneration remains one of the most challenging targets for restorative neuroengineering. When photoreceptors are extensively lost, conventional pharmacological or optical correction is no longer sufficient, and the problem becomes one of restoring an information-bearing interface to the nervous system. Over the last two decades, this landscape has expanded from gene and cell therapies to optogenetics and electronic neuroprostheses, with the unifying goal of reintroducing structured visual information into the surviving retinal or central pathways [1]. Yet, across these approaches, one practical bottleneck repeatedly emerges: even if an intervention can elicit visual percepts, the system-level utility depends on how reliably, safely, and consistently those percepts can support behavior under the variability of real environments [1,2].

Electronic visual prostheses operate this challenge in a concrete way. They transform visual input into stimulation patterns that evoke discrete percepts—commonly described as “phosphenes”—which the user must interpret as functional vision. Modern systems differ by stimulation site (epiretinal, subretinal, suprachoroidal, optic nerve, thalamic, or visual cortex), electrode technology, and coding strategy, but they share a common requirement: an *encoding scheme* must map a high-dimensional, continuously varying scene into a constrained stimulation space while remaining within safety bounds and maintaining perceptual coherence over time [1,2,3]. Consequently, progress in hardware alone does not guarantee progress in usable vision; rather, the choice of encoding and operating policy increasingly governs the realized performance envelope of a device [1,2,3,4].

Clinical and translational milestones in retinal prostheses illustrate both the promise and the limitations of a primarily “function-oriented” development trajectory. The Argus II epiretinal prosthesis, for example, provided clear evidence that electrically evoked percepts can support meaningful tasks (e.g., spatial localization, motion perception, and aspects of reading) in profoundly blind participants, and that these benefits can persist longitudinally [5,6]. Parallel efforts in subretinal systems demonstrated that placing stimulation closer to inner retinal circuitry can yield improved spatial structure under certain conditions, while also introducing different constraints in device integration and long-term stability [7,8,9]. Suprachoroidal approaches further broadened the design space by offering alternative surgical accessibility and electrode placement trade-offs, again emphasizing that “where” and “how” one stimulates is inseparable from what can be encoded and safely delivered [10]. Collectively, these efforts support the feasibility of prosthetic vision but also underscore a core fact: behavioral gains are typically partial, condition-dependent, and strongly mediated by the interaction between stimulation constraints and coding strategies [1,2,3,5,6,7,8,9,10].

Beyond the retina, cortical visual prostheses (VCPs) are re-emerging as a serious candidate for restoring form vision in cases where retinal pathways are no longer viable. A key conceptual advantage is that stimulation can, in principle, access later stages of visual processing even when retinal tissue is irreparably damaged. Recent demonstrations have advanced the field by showing that patterned stimulation—particularly *dynamic* *stimulation strategies* that “trace” shapes across retinotopic cortex—can produce recognizable forms in both sighted and blind humans, challenging the long-standing assumption that cortical prostheses must emulate a static pixel grid [11]. Complementing this, chronic intracortical microelectrode array implantation in a blind participant has shown that multi-electrode-stimulation can evoke discriminable phosphenes with stable thresholds over months, providing important evidence toward the safety and practicality of high-channel-count intracortical interfaces [12]. At the same time, fundamental limitations remain: the relationship between stimulation current and perceived phosphene size can saturate, and phosphene geometry can depend strongly on cortical location and the spread of activation, implying hard constraints on the achievable “resolution” and on the smoothness of perceptual control [13]. These realities motivate a shift in emphasis from single-point performance reporting toward systematic characterization of operating limits and failure modes.

A central reason this shift is necessary is that the *evaluation* *target itself* is structurally multi-dimensional. In many prosthetic vision studies, the primary outcome is task performance—accuracy, completion time, or recognition rate—measured under a small number of experimental conditions. This approach is historically sensible: it aligns with functional goals and yields interpretable endpoints. However, it can obscure two failure channels that are particularly consequential in neuroprosthetics. First, safety constraints are not merely binary “pass/fail” checks; they can compress the feasible stimulation space and force qualitatively different encoding behavior as parameters approach limits. Second, even when outward task performance remains stable, the internal decoder-latent representation structure induced by an encoding scheme may degrade in subtle ways that foreshadow abrupt collapse under additional perturbations. If evaluation remains restricted to a few nominal conditions, such representation-level sensitivities may not be detected until they appear in deployment.

Safety constraints are foundational because electrical stimulation can damage tissue if charge delivery exceeds thresholds that depend on electrode geometry, materials, pulse parameters, and the tissue microenvironment [14,15,16,17]. Classical work proposed empirical safety models relating charge density and charge per phase to damage risk, and later experiments demonstrated that these variables interact in determining neural injury thresholds [14,15]. Engineering reviews have emphasized that safe stimulation is shaped not only by pulse amplitude but also by waveform, electrode polarization dynamics, and material-dependent electrochemistry—meaning that “safe” is inherently a function of both hardware and operating policy [16,17]. Moreover, chronic implants are not static: encapsulation tissue and foreign-body responses can change local electrical properties, reshaping current spread and effective thresholds over time [18]. Modeling studies have likewise shown that tissue conductivity and anisotropy can substantially alter the field distribution that drives neural excitation, reinforcing that safety margins and efficacy are inseparable from biophysical context [19]. In practical terms, these observations imply that an encoding strategy cannot be evaluated solely by performance at a nominal operating point; it must also be characterized by how it behaves as stimulation budgets tighten or as the effective interface drifts.

Perceptual variability further strengthens the case for evaluation beyond average task scores. Psychophysical work in retinal stimulation has demonstrated that stimulation parameters can differentially control percept attributes such as brightness and size, and that amplitude modulation and frequency modulation can have separable effects on the perceived phosphene [20]. Early surgical trials similarly highlighted variability in percept concordance and reproducibility across subjects and electrodes, signaling that the mapping from stimulation to percept is neither perfectly stable nor universally transferable [21]. In cortical stimulation, variability arises not only from electrode placement and cortical magnification but also from the complex dynamics of local circuits and the spread of activation, which can constrain the precision with which percepts can be shaped [13,22]. These realities motivate evaluation frameworks that explicitly represent uncertainty, drift, and condition-dependence, rather than implicitly assuming fixed stimulus–percept mapping.

Simulated prosthetic vision (SPV) plays an enabling role in this methodological shift because it supports controlled, repeatable exploration of encoding behaviors over wide perturbation spaces. SPV frameworks model phosphenes and their composition into a functional scene, allowing systematic manipulation of resolution, dropout, distortion, noise, and luminance quantization in a way that is impractical or unsafe in implanted subjects [23,24]. Importantly, SPV is not merely a convenience; it is a necessary intermediate step for developing evaluation concepts that can later translate to clinical settings. For instance, Chen and colleagues emphasized that simulation studies must be consistent with reported phosphene phenomenology if their conclusions are to generalize to implant recipients, and they argued for standardized simulation and reporting to enable meaningful cross-study comparison [23,24]. This directly aligns with the present work’s focus: evaluation should not be an ad hoc set of case studies, but a structured methodology capable of revealing systematic vulnerabilities and supporting principled operating-point selection.

A productive way to formalize this need is to treat evaluation as *stress* *testing* across a parameterized perturbation space. In engineering disciplines, stress testing refers to systematically exposing a system to controlled degradations to reveal failure thresholds, non-linearities, and hidden fragilities. In the context of prosthetic vision, stressors naturally include phosphene dropout, spatial distortion of the phosphene map, contrast compression, sensor noise, motion-related misalignment, and resource constraints that reduce available stimulation budgets. Critically, these stressors can interact: an encoding scheme that is comparatively resilient under mild dropout may fail catastrophically when dropout co-occurs with luminance reduction; a scheme that preserves average accuracy may exhibit large variance across perturbation realizations, undermining reliability in safety-critical use. Therefore, what matters is not only mean performance but the *shape* of the degradation curve and the presence of abrupt transition regions.

The machine learning robustness literature provides a complementary perspective on why such stress testing is essential. It is now well-established that models can achieve high test accuracy under nominal conditions while being highly sensitive to structured perturbations or distribution shifts. Adversarial examples demonstrate that small but targeted input changes can produce disproportionate failures in otherwise accurate systems [25,26]. Broader analyses of dataset shift and “corruption” benchmarks show that performance can degrade sharply under realistic perturbations not represented in training data, and that “robustness” cannot be inferred from accuracy alone [27,28]. Moreover, reliability under shift is tied to uncertainty estimation and calibration: two models with similar accuracy may differ significantly in their ability to signal when they are likely wrong, affecting downstream decision safety [29]. While prosthetic vision is not a classification benchmark, the underlying lesson generalizes: an encoding strategy evaluated only at a small number of nominal conditions can appear satisfactory while harboring reduced-coupling behavior that becomes operationally significant under realistic variability.

These insights suggest that prosthetic vision evaluation should move from “single-metric comparison” toward “operating-envelope characterization.” This requires (i) parameterizing stress severity and sweeping it systematically, (ii) quantifying not only task performance but also the safety margin under the induced stimulation demands, and (iii) introducing a topology-based representation-metric axis to detect early structural degradation that may precede visible performance loss.

The representation-level axis is motivated by the observation—common in modern systems analysis—that internal representations can drift or collapse in ways that do not immediately manifest in final outputs. Tools for comparing representations, such as singular vector CCA and related approaches, were introduced to study deep network dynamics and interpretability, precisely because test accuracy can be insensitive to substantial internal change [30]. Centered kernel alignment (CKA) and related similarity indices further provide stable measures of representational correspondence across conditions and models, with practical advantages in diagnosing “hidden” changes not evident from output metrics alone [31]. Translating this idea to prosthetic encoding suggests a concrete hypothesis: under increasing stress severity, an encoding scheme may maintain task performance up to a point while its induced decoder-latent representation structure becomes progressively less consistent, creating a topology-sensitive operating region where the system is superficially functional but structurally altered.

From an engineering decision standpoint, the consequence is that encoding evaluation becomes inherently multi-objective. Functional performance remains a primary objective, but it must be considered jointly with safety and topology-based representation metrics. Safety is not merely a constraint that is either satisfied or violated; rather, one must account for *how* *close* the system operates to safety limits and how that proximity changes across stress conditions. Likewise, topology-based representation metrics can be treated as an additional comparative analysis axis within the present framework: a scheme that achieves high accuracy by producing brittle, highly condition-specific representations may be undesirable compared to a slightly less accurate scheme that remains more consistent and less condition-specific under perturbation. This framing naturally leads to Pareto trade-offs and to the need for principled operating-point selection methods, which are well-studied in multi-objective optimization [32]. Furthermore, because stress testing emphasizes tail behavior (rare but consequential failures), risk-aware summaries such as conditional value-at-risk (CVaR) provide a coherent way to prioritize stress resilience against worst-case regions rather than average-case performance [33].

The present study is positioned precisely at this methodological intersection. Rather than proposing yet another encoding algorithm optimized for a single benchmark, we propose an evaluation framework that (i) constructs a structured stress-sweep protocol over perturbation severity, (ii) quantifies three coupled axes—task performance, residual proxy safety burden, and topology-based representation metrics—across that protocol, and (iii) aggregates these trajectories into decision-ready summaries that enable operating-point selection. By design, this framework is meant to reveal counterexamples that conventional evaluations miss: cases where two schemes match in nominal accuracy yet diverge sharply in residual proxy safety-burden profile or in topology-based representation metrics under stress, and cases where representation-level variation becomes more pronounced before comparably large performance degradation, suggesting an additional representation-level diagnostic signal. In doing so, the work reframes phosphene-based encoding assessment as an operating-envelope and risk-management problem, aligning the proposed evaluation methodology with exploratory analysis of stress-sensitive operating conditions in simulated neuroprosthetic-vision settings. At the same time, the present study does not attempt to model physiologically calibrated retinal or cortical phosphene generation. The proposed framework should therefore be read as a controlled simulation-level assessment of encoder behavior under a simplified SPV percept-synthesis operator, not as a subject-specific or implant-calibrated prosthetic-vision simulator.

In this work, we propose a stress-based evaluation framework that systematically characterizes phosphene encoding behavior across controlled perturbation severity. It integrates three coupled axes—functional performance, residual proxy safety burden, and topology-based metrics—within a unified operating-envelope formulation. This formulation enables the identification of reduced coupling regimes between topology-based representation metrics and output-level performance and supports principled operating-point selection beyond conventional single-metric evaluation. Accordingly, encoder preferences reported in this study are valid only within the simplified percept-synthesis and fixed-observer evaluation setting used here.

The remainder of this paper is organized as follows. Section 2 reviews prior work in functional SPV evaluation, stimulation safety and policy constraints, and emerging directions relevant to robustness and representation analysis. Section 3 formalizes the evaluation problem as a multi-objective operating-point selection task under structured stress conditions. Section 4 describes the proposed methodology and the implementation details of the stress model, residual proxy safety-burden metrics, and topology-based representation metrics. Section 5 reports results across representative encoding schemes, highlighting trade-offs, stress-sensitive operating regions, and decision implications. Section 6 concludes with limitations and future directions toward integrating the framework with closed-loop personalization and clinical calibration.

## 2. Related Works

### 2.1. Functional Performance-Oriented Evaluation in Simulated Prosthetic Vision

Visual neuroprosthetics research has historically framed the evaluation of encoding and stimulation strategies primarily in terms of functional task performance. Clinical demonstrations of retinal prostheses such as the Argus II system have provided empirical evidence that even under severe spatial and contrast limitations, electrically evoked phosphenes can support letter and word reading, as well as long-term functional use in daily life contexts [34]. In these studies, system ON versus OFF comparisons were used to quantify improvements in reading accuracy and task execution, thereby establishing functional metrics—rather than purely perceptual similarity—as the principal endpoint. This clinical orientation toward task performance has strongly influenced subsequent simulated prosthetic vision (SPV) research, where invasive stimulation is replaced by controlled visual substitution paradigms in sighted participants.

Within SPV paradigms, researchers have typically manipulated phosphene resolution, spatial arrangement, luminance quantization, dropout rates, and noise characteristics, and subsequently measured task-level performance such as navigation time, obstacle avoidance accuracy, recognition rates, and reading speed. Wang et al. [35], for example, investigated virtual wayfinding under gaze-locked SPV conditions by systematically varying contrast, background noise, and phosphene dropout. Their study indicated that even moderate degradation in contrast or the introduction of dropout could disproportionately increase navigation errors and completion time. Importantly, the study showed that task performance is not a monotonic function of resolution alone; rather, specific degradation modes induce qualitatively distinct failure patterns. This observation suggests that evaluating encoding schemes solely at a single operating point may obscure condition-dependent vulnerabilities.

Similarly, Vergnieux et al. [36] compared conventional phosphene renderings with structure-enhanced renderings that emphasized environmental boundaries and navigational cues. Their work demonstrated that highlighting structural features improved wayfinding efficiency and reduced cognitive load, as inferred from both objective performance metrics and subjective ratings. The implication of this study is that encoding is not merely a matter of spatial sampling density but of semantic structuring. However, while the performance gains were statistically significant under the tested conditions, the robustness of these gains under broader environmental variability was not fully characterized. The evaluation focused primarily on average improvements, leaving open the question of how such encoding strategies behave under systematic stress conditions such as extreme luminance reduction or partial phosphene map distortion.

Beyond navigation tasks, reading performance has served as a canonical functional benchmark. Vurro et al. [37] simulated thalamic prosthetic vision and measured reading accuracy, speed, and acuity across different phosphene configurations. Their results indicated that performance degrades gracefully as spatial sampling decreases, but that specific spatial distortions can produce abrupt reading failures. Notably, the evaluation framework emphasized performance endpoints but did not explicitly model safety constraints or topology-based representation variation. In clinical contexts, da Cruz et al. [34] reported that Argus II recipients achieved measurable reading capability and object recognition improvements over extended follow-up periods, thereby demonstrating translational feasibility. Yet even in these clinical studies, performance was the dominant metric; systematic exploration of operating limits in terms of stimulation safety or representational degradation was not the primary focus.

More recently, Ho et al. [38] employed augmented-reality-based SPV to approximate real-world tasks, including object recognition and mobility under variable field-of-view and grayscale quantization constraints. Their methodology emphasized ecological validity, moving beyond laboratory-based static images. The results suggested that task complexity interacts nonlinearly with spatial resolution and contrast depth. While this work significantly advanced the realism of SPV evaluation, the evaluation criteria remained performance-centric, without a unified framework to simultaneously evaluate safety margins or representation-level consistency across perturbations.

Collectively, the performance-oriented literature establishes that encoding schemes materially influence functional outcomes and that degradation modes can have nontrivial behavioral consequences. However, the predominant evaluation paradigm compares average task performance across a limited set of conditions. Systematic exploration of multidimensional perturbation spaces—combined with explicit modeling of safety and topology-based representation variation—remains comparatively underdeveloped.

### 2.2. Safety Constraints and Stimulation Policies

Electrical stimulation safety is foundational in neuroprosthetics, as stimulation parameters must remain within limits that prevent tissue damage. Early experimental studies demonstrated that both charge density and charge per phase contribute to neural injury risk, and that their interaction must be considered when defining safe stimulation boundaries [15]. These findings were complemented by theoretical safety models such as Shannon’s formulation [14], which proposed empirical relationships between charge density, phase duration, and tissue damage risk. Cogan et al. [39] further reviewed tissue damage thresholds in therapeutic electrical stimulation, emphasizing that safe operating regimes depend on electrode material, geometry, and pulse characteristics.

In the context of retinal prostheses, these safety constraints manifest operationally in the need to limit simultaneous electrode activation and cumulative charge injection. Kasowski et al. [40] investigated rastering strategies designed to reduce instantaneous current load by temporally distributing electrode activation. Their results showed that a checkerboard raster pattern preserved object recognition performance while reducing potential overstimulation risk. This study represents an important bridge between safety constraints and functional performance: safety is not treated merely as a post hoc filter but as a design variable shaping stimulation policy.

Nonetheless, even in this line of work, safety is primarily considered as a constraint that must be satisfied while maximizing task performance. The evaluation framework does not typically treat safety as a graded objective whose margin of violation can be mapped continuously across stress conditions. Nor is safety jointly analyzed with topology-based representation variation, i.e., whether approaching safety limits produces subtle distortions in decoder-latent representation structure prior to overt performance decline. As such, while safety-aware optimization is emerging, a unified evaluation methodology that visualizes the interplay between performance, safety margin, and topology-based representation variation remains lacking.

### 2.3. Personalization, Eye Movements, and Calibration

Individual variability in phosphene perception presents an additional layer of complexity. Titchener et al. [41] demonstrated that phosphene locations in suprachoroidal implant users can be estimated using eye-movement-based calibration techniques. Their findings suggest that perceptual maps differ significantly between individuals and may drift over time. This underscores that encoding strategies cannot be evaluated independently of personalized calibration.

Similarly, gaze-contingent processing has been shown to improve mobility and scene recognition under head-steered SPV conditions [42]. By updating phosphene patterns according to gaze direction, the study mitigated spatial updating conflicts and improved subjective perceptual quality. This indicates that encoding performance is contextually embedded within sensorimotor loops rather than static input-output transformations.

However, personalization and gaze-contingent strategies are often incorporated as preprocessing steps rather than integrated into a comprehensive evaluation framework. The interaction between personalization, safety limits, and stress-induced degradation has not been systematically quantified. Consequently, although personalization enhances practical performance, its influence on stability under perturbation and safety boundary conditions remains insufficiently characterized.

### 2.4. Simulation and Differentiable Optimization Frameworks

Advances in computational modeling have facilitated in silico experimentation with encoding and stimulation strategies. The pulse2percept framework [43] provides a modular simulation environment for modeling retinal implant percepts, enabling reproducible comparisons across encoding algorithms. More recently, van der Grinten et al. [44] introduced a biologically plausible, differentiable phosphene simulator that permits gradient-based optimization of stimulation parameters. This development enables end-to-end learning of encoding strategies tailored to specific perceptual objectives.

These frameworks substantially enhance methodological rigor and reproducibility. However, their evaluation outputs remain predominantly tied to task accuracy or perceptual similarity metrics. The differentiable nature of the simulator supports optimization but does not inherently define the evaluation criteria for robustness or safety-aware trade-offs. In other words, while optimization capacity has expanded, the conceptual definition of “optimal” remains largely performance driven.

### 2.5. Topology-Based Representation Analysis as an Underexplored Axis

In machine learning and systems neuroscience, representation-level analysis has become central to understanding stress-response behavior and generalization. Representational Similarity Analysis (RSA) quantifies condition-dependent pattern similarity [45], and Centered Kernel Alignment (CKA) offers a stable measure of representational correspondence across neural networks or layers [31]. Persistent homology-based topological metrics provide complementary insight into structural changes in high-dimensional embeddings [46].

Despite their relevance, such representation-level analyses are rarely incorporated into SPV evaluation. Most studies implicitly assume that if task accuracy remains stable, internal representations are adequate. Yet stress- robustness literature in machine learning has shown that representation degradation can precede performance collapse. Applying analogous diagnostics to prosthetic encoding could reveal early warning indicators of failure under stress conditions.

### 2.6. Positioning of the Present Study

In summary, the related literature has advanced along several important axes: (i) functional task performance benchmarking [35,36,37,38]; (ii) safety-aware stimulation policy design [14,15,39,40]; (iii) personalization and gaze integration [41,42]; and (iv) simulation-enabled optimization [43,44]. Nevertheless, these axes remain largely compartmentalized. No unified framework explicitly models encoding evaluation as a tri-objective problem in which functional performance, residual proxy safety burden, and topology-based representation metrics are jointly quantified across systematically constructed stress conditions.

The absence of such integration limits the ability to identify counterexamples in which two encoding schemes exhibit comparable performance yet diverge significantly in residual proxy safety-burden profile or representation-level structural behavior under stress. It also impedes principled operating-point selection in safety-critical neuroprosthetic systems. The present study addresses this methodological gap by proposing a structured stress-testing paradigm in which perturbation severity is parameterized, residual post-projection proxy burden is quantified, and topology-based representation metrics are incorporated alongside task performance. Through this integration, encoding evaluation is reframed as a multi-objective decision problem rather than a single-metric comparison.

**Figure 1:**
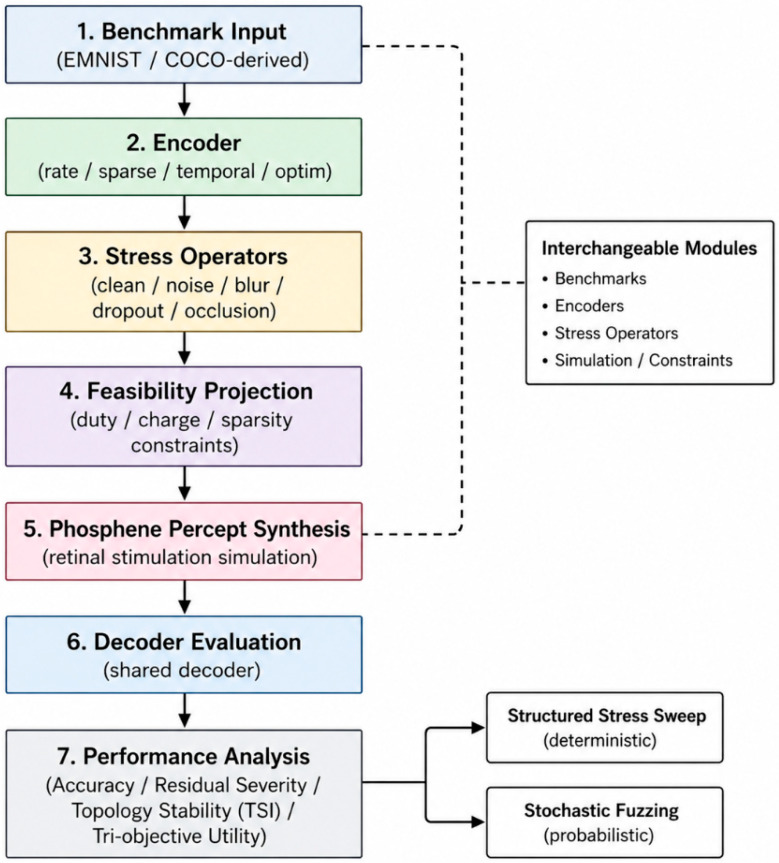
Overview of the proposed stress-based phosphene-encoder evaluation framework. The pipeline consists of modular benchmark, encoding, degradation, feasibility-projection, percept-synthesis, decoder-evaluation, and multi-objective analysis stages. The framework supports structured stress sweeps and stochastic fuzzing analysis under interchangeable encoder and perturbation configurations.

Figure 1 summarizes the overall structure of the proposed stress-based phosphene-encoder evaluation framework. The pipeline is organized as a sequence of modular processing stages consisting of benchmark input, phosphene encoding, stress degradation, feasibility projection, percept synthesis, decoder evaluation, and multi-objective performance analysis. The modular design allows benchmark datasets, encoder implementations, stress operators, and feasibility constraints to be replaced or reconfigured independently while preserving a common evaluation protocol. In addition to deterministic structured stress sweeps, the framework also supports stochastic fuzzing analysis for identifying concentrated stress-sensitive operating regions and representative counterexamples. Accordingly, the present framework should be interpreted as a framework demonstration under simplified SPV conditions intended for controlled comparative stress analysis rather than as a clinically or physiologically realistic prosthetic-vision simulation.

## 3. Problem Formulation

This study reformulates the evaluation of phosphene-encoding schemes not as a single-metric comparison, but as a multi-objective operating-envelope analysis under structured stress. Because the released implementation evaluates multiple benchmark tasks and trains a benchmark-specific shared decoder within each benchmark, the mathematical formulation is written here in a benchmark-aware form. The objective is to quantify how an encoder behaves across three coupled axes—functional performance, residual proxy safety burden, and topology-based representation metrics—under controlled perturbations.

### 3.1. Benchmark-Aware Encoding Model

Let B denote the set of benchmark tasks. In the present implementation,

(1) B={EMNIST Letters, COCO−derived 4−class subset}.

For each benchmark b∈B, let the input space be

(2) $$X^{\left(b\right)}\subset R^{H\times W},$$

and let $Y^{\left(b\right)}$ denote the corresponding label space. An encoding scheme is defined as an operator

(3) $$E_{\theta }:X^{\left(b\right)}\to U,\;\;U\subset R^{N_{e}\times T},$$

where θ∈Θ denotes encoder identity, $N_{e}$ is the number of electrodes, and T is the stimulation sequence length or operating-time parameterization.

A fixed phosphene simulator (or percept-synthesis operator) Φ maps stimulation to a percept representation:

(4) $$Z_{\theta }^{\left(b\right)}(X)=\Phi (E_{\theta }(X)).$$

The present Φ operator is intended as a simplified SPV generation operator for controlled comparative stress analysis and does not constitute a biologically faithful retinal or cortical phosphene simulator. Accordingly, all percept-level outputs in this study should be interpreted as simplified, reproducible SPV representations for comparative stress analysis, not as physiologically calibrated predictions of patient-specific retinal or cortical phosphene percepts. It does not model physiologically calibrated phosphene generation, subject-specific phosphene maps, implant-specific electrode geometry, current spread, or temporal percept dynamics. Because the released implementation trains one shared decoder **per benchmark**, the decoder is indexed by b:

(5) $$D_{b}:Z\to Y^{\left(b\right)}.$$

The benchmark-specific prediction pipeline is therefore

(6) $$f_{\theta }^{\left(b\right)}(X)=(D_{b}\circ \Phi \circ E_{\theta })(X).$$

Within each benchmark, the same decoder $D_{b}$ is shared across all encoders and is held fixed after training. This design removes decoder retraining as an experimental variable and provides a shared downstream observer across encoders. However, stressed performance should still be interpreted as a joint encoder–decoder response under the fixed-observer setting rather than as a fully disentangled encoder-only effect.

### 3.2. Structured Stress Model with Operator Index

To move beyond clean-condition evaluation, we define a structured stress condition with a pair

(7) (o, α),

where o∈O denotes a stress operator and α∈A denotes stress level. In the released implementation,

(8) O={clean,noise,blur,dropout,occlusion},

and

(9) A={0.00, 0.25, 0.50, 0.75, 1.00}.

For each operator o, let

(10) $$T_{\alpha }^{o}:X^{\left(b\right)}\to X^{\left(b\right)}$$

denote the corresponding image-level stress transform. After the stress transform, the encoder output is passed through a feasibility projection $\Pi_{safe}$ that enforces the operating constraints:

(11) $$U_{\theta ,o,\alpha }^{\left(b\right)}(X)=\Pi_{safe}\left(E_{\theta }(T_{\alpha }^{o}(X))\right).$$

The associated percept representation is

(12) $$Z_{\theta ,o,\alpha }^{\left(b\right)}(X)=\Phi \left(U_{\theta ,o,\alpha }^{\left(b\right)}(X)\right),$$

and the stressed prediction pipeline becomes

(13) $$f_{\theta ,o,\alpha }^{\left(b\right)}(X)=D_{b}\left(Z_{\theta ,o,\alpha }^{\left(b\right)}(X)\right).$$

This formulation distinguishes three sources of variation:

1. Benchmark b, which determines the task and label space.
2. Encoder θ, which determines how the image is converted into stimulation.
3. Stress condition (o,α), which determines how the input is perturbed before encoding.

The feasibility projection was designed to represent simplified but physiologically motivated stimulation-control constraints commonly discussed in retinal and cortical prosthesis research. In the present framework, the duty-related constraint discourages excessive temporal stimulation concentration, whereas the proxy-charge constraint approximates the need to avoid disproportionately large cumulative stimulation burden across activated phosphene channels. The sparsity-related constraint penalizes unrealistically dense simultaneous activation patterns that may reduce perceptual separability and increase stimulation overhead. The selected constraint magnitudes should therefore be interpreted as normalized feasibility-control parameters for comparative stress analysis rather than device-specific clinical thresholds. These quantities are therefore used as proxy feasibility-control terms rather than direct clinical or electrochemical safety measurements.

### 3.3. Functional Performance

For a given benchmark b, encoder θ, operator o, and stress level α, functional performance is defined as

(14) $$P^{\left(b\right)}(\theta ,o,\alpha )=E_{(X,Y)∼D^{\left(b\right)}}\left[I\left(f_{\theta ,o,\alpha }^{\left(b\right)}(X)=Y\right)\right].$$

In empirical evaluation, this corresponds to benchmark-specific classification accuracy on the test split:

(15) $$P^{\left(b\right)}\left(\theta ,o,\alpha \right)=\frac{1}{N_{b}}\sum_{i=1}^{N_{b}}I\left(D_{b}\left(Z_{\theta ,o,\alpha }^{\left(b\right)}\left(X_{i}\right)\right)=Y_{i}\right).$$

Thus, functional performance is evaluated not only at the clean condition (clean, 0), but also across the full structured set of stress operators and levels.

### 3.4. Residual Proxy Safety Burden and Integrated Risk Summary

Let $C\left(U_{\theta ,o,\alpha }^{\left(b\right)}(X)\right)$ denote a stimulation-budget functional derived from the projected stimulus. In the released implementation, this burden is represented through three normalized residual post-projection violation terms associated with duty-cycle, proxy charge, and sparsity constraints. Their maximum defines the scalar residual severity:

(16) $${sev}^{\left(b\right)}(\theta ,o,\alpha ;X)=max\left({sev}_{duty},{sev}_{charge},{sev}_{sparse}\right).$$

At the condition level, the implementation reports the worst-case residual severity over the benchmark test split:

(17) $${sev}_{max}^{\left(b\right)}(\theta ,o,\alpha )=\underset{i}{max}{sev}^{\left(b\right)}(\theta ,o,\alpha ;X_{i}).$$

This quantity should be interpreted as a **residual proxy safety burden after feasibility projection**, not as a raw pre-projection violation. The selected feasibility constraints were designed to represent simplified but physiologically motivated stimulation-control limits commonly discussed in retinal and cortical prosthesis research. The duty-related constraint was intended to discourage excessive temporal stimulation concentration, whereas the proxy-charge constraint approximated the need to avoid disproportionately large cumulative stimulation burden across activated phosphene channels. The sparsity-related constraint was introduced to penalize unrealistically dense simultaneous activation patterns that may reduce perceptual separability and increase stimulation overhead. The absolute values used in the present simulations should therefore be interpreted as normalized feasibility-control parameters for comparative stress analysis rather than device-specific clinical thresholds. For this reason, the residual proxy safety-burden values reported in this study should not be interpreted as electrochemical tissue-damage estimates, clinical safety margins, or implant-specific stimulation-safety guarantees. They are used only as normalized comparative indicators of post-projection feasibility burden within the present simplified simulation framework.

To summarize residual proxy safety burden over the structured stress grid, we define the operator-averaged severity at each stress level:

(18) $${\bar{sev}}_{max}^{\left(b\right)}(\theta ,\alpha )=\frac{1}{\left|O^{*}\right|}\sum_{o\in O^{*}}{sev}_{max}^{\left(b\right)}(\theta ,o,\alpha ),$$

where

(19) $$O^{*}=\{\mathrm{noise},\mathrm{blur},\mathrm{dropout},\mathrm{occlusion}\}$$

excludes the clean condition.

Using the discrete stress grid spacing Δα, the integrated residual proxy safety burden is

(20) $$R_{safe}^{\left(b\right)}\left(\theta \right)=\sum_{\alpha \in A^{*}}{\bar{sev}}_{max}^{\left(b\right)}\left(\theta ,\alpha \right)\;\Delta \alpha ,$$

where $A^{*}=\{0.25,0.50,0.75,1.00\}$ or, more generally, the non-clean stress levels used in the implementation summary.

### 3.5. Topology-Based Representation Metrics

Even when task performance remains acceptable, the percept-induced internal representation may become structurally altered under stress. To quantify this effect within the present exploratory framework, we define a topology-based representation metric.

This metric is intended as an exploratory and configuration-dependent topology-based representation diagnostic derived from topological deviation relative to the clean condition. It should not be interpreted as a validated general measure of latent representational stability. Larger values indicate smaller topological deviation from the clean-condition latent point cloud under the specific PCA, subset-size, and persistent-homology configuration used in this study. To quantify this effect, we define a topology-based representation metric in the decoder-latent space. For each benchmark b, encoder θ, operator o, and stress level α, let

(21) $$z_{i}^{\left(b\right)}(\theta ,o,\alpha )\in R^{d_{0}}$$

denote the latent representation of sample $X_{i}$ extracted from the penultimate layer of the frozen benchmark-specific decoder $D_{b}$.

Using a fixed embedding map $\psi_{\theta }^{\left(b\right)}$ learned from the clean condition, we form the projected point cloud

(22) $$P_{\theta ,o,\alpha }^{\left(b\right)}={\left\{\psi_{\theta }^{\left(b\right)}\left(z_{i}^{\left(b\right)}(\theta ,o,\alpha )\right)\right\}}_{i=1}^{N_{TDA}}\subset R^{d}.$$

Persistent homology is then computed on this point cloud to produce a persistence diagram

(23) $$D_{\theta ,o,\alpha }^{\left(b\right)}.$$

The topological deviation from the clean condition is defined as

(24) $$d_{topo}^{\left(b\right)}(\theta ,o,\alpha )=Dist\left(D_{\theta ,\mathrm{clean},0}^{\left(b\right)},D_{\theta ,o,\alpha }^{\left(b\right)}\right).$$

Using a robust encoder-specific scale parameter $\tau_{\theta }^{\left(b\right)}$, the normalized topology-based representation diagnostic index is

(25) $$R^{\left(b\right)}(\theta ,o,\alpha )=exp\left(-\frac{d_{topo}^{\left(b\right)}(\theta ,o,\alpha )}{\tau_{\theta }^{\left(b\right)}}\right),\;\;R^{\left(b\right)}(\theta ,o,\alpha )\in (0,1].$$

Thus, larger values of $R^{\left(b\right)}$ indicate smaller topology-based deviation from the clean-condition latent point cloud under the specific PCA, subset-size, and persistent-homology configuration used in this study.

### 3.6. Reduced-Coupling Region

A key concept in this study is the reduced-coupling region between topology-based representation metrics and output-level performance under stress, namely the subset of stress conditions for which task performance remains relatively preserved while topology-based representation metrics have already changed substantially.

For benchmark b and encoder θ, define

(26) $$L_{\theta }^{\left(b\right)}=\left\{(o,\alpha )\;\;R^{\left(b\right)}(\theta ,o,\alpha )<\tau_{R},\;P^{\left(b\right)}(\theta ,o,\alpha )>\tau_{P}\right\},$$

where $\tau_{R}$ and $\tau_{P}$ are user-defined topology-based representation-metric and performance thresholds.

If $L_{\theta }^{\left(b\right)}\neq ⌀$, then the encoder exhibits a regime in which large output-level degradation has not yet occurred, but the topology-based representation metric has already decreased substantially. This region is operationally important because it cannot be detected by performance-only evaluation.

### 3.7. Integrated Tri-Objective Evaluation

To summarize encoder behavior over the structured stress grid, we first define operator-balanced level-wise means for functional performance and topology-based representation metrics:

(27) $${\bar{P}}^{\left(b\right)}(\theta ,\alpha )=\frac{1}{\left|O^{*}\right|}\sum_{o\in O^{*}}P^{\left(b\right)}(\theta ,o,\alpha ),$$

(28) $${\bar{R}}^{\left(b\right)}(\theta ,\alpha )=\frac{1}{\left|O^{*}\right|}\sum_{o\in O^{*}}R^{\left(b\right)}(\theta ,o,\alpha ).$$

Using the discrete stress spacing Δα, we define

(29) $${TSI}_{P}^{\left(b\right)}(\theta )=\sum_{\alpha \in A^{*}}{\bar{P}}^{\left(b\right)}(\theta ,\alpha )\;\Delta \alpha ,$$

(30) $${TSI}_{R}^{\left(b\right)}(\theta )=\sum_{\alpha \in A^{*}}{\bar{R}}^{\left(b\right)}(\theta ,\alpha )\;\Delta \alpha .$$

Together with the integrated residual proxy safety burden $R_{safe}^{\left(b\right)}(\theta )$, these define the benchmark-specific tri-objective utility:

(31) $$U^{\left(b\right)}(\theta )=\lambda_{P}{TSI}_{P}^{\left(b\right)}(\theta )+\lambda_{R}{TSI}_{R}^{\left(b\right)}(\theta )-\lambda_{S}R_{safe}^{\left(b\right)}(\theta ).$$

In the released implementation,

(32) $$\lambda_{P}=\lambda_{R}=\lambda_{S}=1.$$

Therefore, the utility reflects an equal-weight compromise among stressed functional performance, representation preservation, and residual proxy safety burden.

### 3.8. Operating-Point Selection

For a fixed benchmark b, the preferred encoder under the implemented evaluation policy is selected as

(33) $$\theta_{b}^{*}=arg\underset{\theta \in \Theta }{max}U^{\left(b\right)}(\theta ).$$

Because the same encoder may not be optimal across all benchmarks, the manuscript-level interpretation is based not only on benchmark-specific utility but also on cross-benchmark comparison of

(34) $${TSI}_{P}^{\left(b\right)},\;{TSI}_{R}^{\left(b\right)},\;R_{safe}^{\left(b\right)},\;U^{\left(b\right)}.$$

Accordingly, the present study does not treat encoder evaluation as a single global ranking problem. Rather, it treats it as an operating-envelope analysis in which encoder preference may depend on the benchmark task, the degradation regime, and the trade-off between functional utility and residual proxy safety burden.

## 4. Materials and Methods

This section translates the formal framework in Section 3 into an executable evaluation protocol implemented in the released FINAL_REPRO_RunPackageC_EMNIST_COCO_seed0042_20260614.ipynb notebook. The final fixed-seed canonical analysis was executed in Python 3.12.13 on Windows 11 using NumPy 2.0.2, pandas 2.2.2, and PyTorch 2.11.0+cpu. All computations were performed on a CPU, and CUDA acceleration was not used. The random seed was fixed at 42 for the final reproducibility run. To avoid conflating methodological definition with empirical findings, this section introduces the benchmark suite, encoding operators, stress model, decoder training protocol, residual proxy safety-burden metrics, topology-based representation analysis, and stochastic fuzzing procedure, while reserving all outcome-level observations for Section 5.

### 4.1. Encoding Schemes

Input images are converted to grayscale, normalized to the range [0,1], and embedded into a fixed square canvas of size 64×64. The released implementation provides both a reduced *pilot* mode and a manuscript-level *final* mode; unless otherwise stated, the methods described below refer to the final configuration used for the main experiments.

To move beyond a toy benchmark and to cover more than one functional axis of simulated prosthetic vision, we employ a two-benchmark suite. The first benchmark is **EMNIST Letters**, used for symbolic recognition, with 26 classes corresponding to uppercase letters. The second benchmark is a **COCO-derived balanced subset**, used for a reduced four-class COCO-derived image-level classification task, with four image-level classes defined according to the presence of the target categories *person* and *car*: none, person, car, and both. For the manuscript-level configuration, EMNIST is evaluated using 20,000/3000/3000 train/validation/test samples, while the COCO-derived benchmark is evaluated using 4000/1000/1000 samples, corresponding to 1000 images per class. All splits are generated under a fixed random seed of 42.

The two benchmarks are constructed differently. EMNIST uses the official train/test partition, and the validation set is obtained by a stratified split of the original training partition. In contrast, the COCO-derived benchmark is assembled from a streamed training partition, from which balanced quotas are sequentially assigned to train, validation, and test subsets. Specifically, a sample is assigned to the label

(35) $$y=\left\{\begin{array}{ll} 0, & \mathrm{if}\;\mathrm{neither}\;\mathrm{person}\;\mathrm{nor}\;\mathrm{car}\;\mathrm{is}\;\mathrm{present};\\ 1, & \mathrm{if}\;\mathrm{person}\;\mathrm{is}\;\mathrm{present}\;\mathrm{but}\;\mathrm{car}\;\mathrm{is}\;\mathrm{absent};\\ 2, & \mathrm{if}\;\mathrm{car}\;\mathrm{is}\;\mathrm{present}\;\mathrm{but}\;\mathrm{person}\;\mathrm{is}\;\mathrm{absent};\\ 3, & \mathrm{if}\;\mathrm{both}\;\mathrm{person}\;\mathrm{and}\;\mathrm{car}\;\mathrm{are}\;\mathrm{present}. \end{array}\right.$$

This design intentionally favors benchmark balance and controlled comparison over adherence to an official COCO classification split.

For both benchmarks, the preprocessed image is first resampled to a fixed 16×16 electrode lattice and then mapped by an encoder $E_{\theta }$ to an electrode-level stimulation pattern. The resulting stimulation is converted into a phosphene-like percept representation by a fixed percept-synthesis operator Φ. This operator was used to provide a controlled and reproducible percept-synthesis stage for comparative stress analysis. It should not be interpreted as a physiologically calibrated retinal or cortical phosphene simulator. In the implementation, Φ upsamples the electrode-amplitude map to a 32×32 percept grid using nearest-neighbor expansion and then applies Gaussian smoothing with σ=1.0. The smoothed percept is additionally scaled by a gain term derived from the global stimulation frequency and pulse width:

(36) $$\Phi (U)=clip\left(Gauss\left(Upsample(A)\right)\cdot \left(0.5+\frac{f}{f_{max}}\frac{pw}{pw_{max}}\right),\;0,\;1\right),$$

where A denotes the electrode-amplitude map, f is the stimulation frequency, and pw is the pulse width. We compare four encoding strategies,

(37) θ∈{rate,sparse,temporal,optim}.

The **rate** encoder performs direct rate-based amplitude mapping. The input image is resampled to the electrode grid, and electrode amplitudes are set proportional to local intensity. The global stimulation frequency f and pulse width pw are determined from the image mean intensity m by

(38) $$f=f_{min}+(f_{max}-f_{min})m,\;\;pw=max\left(pw_{min},min\left(pw_{max},\frac{duty_{max}}{f}\right)\right).$$

The **sparse** encoder first ranks electrode candidates by local intensity and then applies a spatial-diversity constraint through greedy top-k selection with Manhattan minimum separation =2. Amplitudes are assigned only to the selected electrodes, after which the same global f–pw policy is used.

The **temporal** encoder implements latency modulation followed by time-weighted integration. Let g be the resampled electrode grid. The active set is defined by the top-$k_{max}$ responses, and the latency map is given by

(39) l=(1−g)⊙M,

where M is the binary active-electrode mask. Effective amplitudes are then obtained by exponential weighting,

(40) $$A\propto exp\left(-\frac{l}{\tau_{t}}\right)⊙M,$$

with $\tau_{t}=0.25$, followed by normalization and global scaling to $0.8\;a_{max}$.

The **optim** encoder solves a simple constrained optimization problem on the electrode grid. Starting from the target grid $x^{\left(0\right)}=g$, the code iteratively applies a quadratic reconstruction step, soft threshold sparsification with λ=0.08, amplitude clipping, and sparsity projection:

(41) $$x^{\left(t+1\right)}=\Pi_{sparse}\left(clip\left(S_{\lambda }\left(x^{\left(t\right)}-\eta \;2(x^{\left(t\right)}-g)\right),0,1\right)\right),$$

with 20 iterations and step size η=0.5.

All encoder outputs are passed through the same feasibility projection before percept synthesis. This step clips amplitudes and operating parameters to predefined bounds, enforces the duty-cycle constraint by shrinking pulse width when necessary, applies sparsity projection to satisfy the active-electrode limit, and rescales amplitudes globally if the proxy charge budget is exceeded. Consequently, encoder comparison is carried out under identical post-encoding feasibility rules, rather than encoder-specific correction policies. Table 1 summarizes the key components of the proposed evaluation framework, including stress operators, metric definitions, and evaluation axes. The configuration summarized in Table 1 establishes a controlled comparison setting across all evaluated encoding strategies. In particular, all encoders were evaluated under identical preprocessing conditions, shared decoder architectures, and common operating constraints in order to support controlled comparison under matched preprocessing, decoder, and operating-constraint assumptions. The two benchmark tasks were intentionally designed to represent different representational regimes, including structured symbolic recognition in EMNIST and compressed low-accuracy image-level classification in the reduced COCO-derived benchmark. Maintaining fixed decoder and evaluation settings across all experiments also reduces the possibility that observed differences originate from task-specific optimization or unequal post-processing conditions. Consequently, the framework enables direct comparison of performance, residual proxy safety burden, and topology-based representation metrics under matched experimental assumptions.

### 4.2. Stress Operator and Severity Definition

Following Section 3.2, stress is implemented directly at the image level before encoding. In the released code, a stress condition is specified by an operator o∈{clean,noise,blur,dropout,occlusion} and a scalar level

(42) α∈{0.00, 0.25, 0.50, 0.75, 1.00}.

The clean condition is included as a reference condition, whereas the four non-clean operators are used to generate degradation curves and stress summaries.

The **Gaussian noise** operator is defined as

(43) $$T_{\alpha }^{noise}\left(X\right)=clip\left(X+\varepsilon ,0,1\right),\;\;\varepsilon ∼N\left(0,\sigma (\alpha {)}^{2}\right),\sigma (\alpha )=0.6\alpha .$$

The **blur** operator applies Gaussian smoothing to the image:

(44) $$T_{\alpha }^{blur}(X)=clip\left(G_{\sigma (\alpha )}\times X,0,1\right),\;\;\sigma (\alpha )=1.5\alpha .$$

The **dropout** operator applies element-wise Bernoulli masking:

(45) $$T_{\alpha }^{dropout}(X)=M_{\alpha }⊙X,\;\;M_{\alpha }(i,j)∼Bernoulli(1-p(\alpha )),p(\alpha )=0.6\alpha .$$

The **occlusion** operator suppresses a contiguous rectangular patch whose side lengths are proportional to the stress level. Let h×w denote the image size. Then, the occlusion patch size is

(46) $$(h_{o},w_{o})=\left(max(1,\lfloor 0.6\alpha h\rfloor ),\;max(1,\lfloor 0.6\alpha w\rfloor )\right),$$

and the patch location is sampled uniformly within the valid image domain.

Stress is evaluated over all encoder-level and operator-level combinations. For each stressed image, the encoder produces a projected stimulus

(47) $$U_{\theta ,o,\alpha }(X)=\Pi_{safe}\left(E_{\theta }(T_{\alpha }^{o}(X))\right),$$

where $\Pi_{safe}$ denotes the feasibility projection described in Section 4.1.

Residual proxy safety burden is quantified through three normalized residual post-projection violation terms computed on the projected stimulus U:

(48) $${sev}_{duty}=max\left(0,\frac{f\cdot pw-duty_{max}}{duty_{max}}\right),$$

(49) $${sev}_{charge}=max\left(0,\frac{\sum A\cdot f\cdot pw-q_{max}}{q_{max}}\right),$$

(50) $${sev}_{sparse}=max\left(0,\frac{n_{active}-k_{max}}{k_{max}}\right).$$

The condition-wise scalar severity used in the exported results is

(51) $$sev=max\left({sev}_{duty},{sev}_{charge},{sev}_{sparse}\right).$$

In the implementation,

(52) $$duty_{max}=0.10,\;\;q_{max}=0.015,\;\;k_{max}=40.$$

Because severity is evaluated after feasibility projection, the resulting quantity should be interpreted as a residual post-projection proxy violation rather than as a raw pre-projection burden or a direct clinical safety measurement. The maximum stress levels were intentionally selected to span the transition from mild perceptual degradation to near-task-collapse conditions while still preserving visually interpretable phosphene structure. Thus, the present stress grid was designed not as a clinical calibration scale, but as a controlled operating-envelope exploration covering progressively adverse yet behaviorally meaningful perturbation regimes.

Detailed stress-operator and sweep-level specifications are provided in the supplementary file Table4_1_BenchmarkConfig.csv, while the residual proxy severity definitions are provided in Table2_SafetyConstraints_SeverityDefinitions.csv.

### 4.3. Decoder Configuration

For fair comparison, the decoder is trained once **within each benchmark** and is then held fixed throughout all subsequent analyses on that benchmark. Thus, the decoder is not retrained per encoder, per stress operator, per stress level, or during fuzzing. This design keeps the downstream observer fixed and reduces confounding variation introduced by repeated decoder retraining.

The decoder is trained on **clean percepts pooled across encoders**. Let b denote a benchmark. For each encoder θ, clean percepts are generated from the benchmark training and validation sets:

(53) $$Z_{tr}^{\left(b\right)}=\underset{\theta \in \Theta }{⋃}\Phi (E_{\theta }(X_{tr}^{\left(b\right)})),\;\;Z_{va}^{\left(b\right)}=\underset{\theta \in \Theta }{⋃}\Phi (E_{\theta }(X_{va}^{\left(b\right)})).$$

The pooled clean percepts are then used to train a benchmark-specific shared decoder $D_{b}$.

The decoder architecture is identical across benchmarks and consists of two convolutional blocks followed by a latent fully connected layer and a final linear classifier:

(54) $$Conv(1\to 16,3)\to ReLU\to MaxPool(2)\to Conv(16\to 32,3)\to ReLU\to MaxPool(2)\to FC(64)\to Linear(n_{classes}).$$

The percept input size is 32×32, the latent dimension is 64, the optimizer is Adam, the learning rate is $10^{-3}$, the batch size is 128, and the number of training epochs is 8. Cross-entropy loss is used throughout. No early stopping or benchmark-specific hyperparameter tuning is applied in the released implementation.

After training, the decoder weights are frozen and reused for all downstream stress sweeps, topology analyses, and fuzzing procedures performed on that benchmark. A benchmark-specific frozen decoder was intentionally maintained across encoder, stress, and fuzzing conditions in order to provide a consistent downstream observer and reduce confounding variation introduced by repeated decoder retraining under heterogeneous stress conditions. However, this fixed-decoder design also limits causal attribution. Because the decoder was not retrained or adapted under stressed percept distributions, the reported performance degradation cannot be fully decomposed into encoder-induced changes and decoder sensitivity to distribution shift. Accordingly, the reported degradation trends should be interpreted as configuration-dependent joint encoder–decoder stress responses under the present fixed-observer protocol rather than as fully disentangled encoder-only robustness measurements.

Because the decoder is benchmark-specific, the notation D in Section 3 should be interpreted here as $D_{b}$, where the benchmark index is suppressed for simplicity unless cross-benchmark comparisons are explicitly discussed.

### 4.4. Functional Performance and Residual Proxy Safety-Burden Evaluation

At each encoder-level and operator-level condition, classification performance is evaluated on the benchmark test split. Let N denote the number of test samples for a given benchmark. Accuracy is computed as

(55) $$P(\theta ,o,\alpha )=\frac{1}{N}\sum_{i=1}^{N}I\left(D_{b}(Z_{\theta ,o,\alpha }(X_{i}))=Y_{i}\right).$$

In addition to performance, the implementation records three residual proxy safety-burden summaries over the same test set. Let ${sev}_{i}$ be the scalar residual severity of the projected stimulus associated with test image $X_{i}$. Then, the code exports

(56) $${sev}_{max}(\theta ,o,\alpha )=\underset{i}{max}{sev}_{i},$$

(57) $${sev}_{mean}(\theta ,o,\alpha )=\frac{1}{N}\sum_{i=1}^{N}{sev}_{i},$$

(58) $$r_{viol}(\theta ,o,\alpha )=\frac{1}{N}\sum_{i=1}^{N}I({sev}_{i}>0).$$

Accordingly, the present implementation distinguishes clearly between (i) condition-wise stress measurements and (ii) stress-range summaries derived from them. This distinction should be preserved in the manuscript so that the clean baseline, degradation curves, and worst-case summaries are not conflated.

Detailed condition-wise functional and residual proxy safety-burden metrics are provided in the supplementary file TableStress_PerfSafety_ByEncoderOpLevel.csv.

### 4.5. Topological Representation Analysis

To quantify topology-based representation metrics under stress, we analyze the latent representations produced by the penultimate decoder layer rather than raw percept images. For a fixed benchmark, encoder, operator, and stress level, let $z_{i}\in R^{64}$ denote the latent representation of test sample $X_{i}$ obtained from the decoder output immediately before the final linear classifier.

For computational control, topology analysis is performed on a fixed subset of test images: $N_{TDA}=min(300,\;N_{test}).$ For each encoder separately, PCA is fitted on the clean latent representations, $Z_{\theta ,clean,0}=\{z_{i}{\}}_{i=1}^{N_{TDA}},$ and all stressed latent sets for that encoder are projected using the same PCA map. The PCA dimension is d=min(10, 64).

Persistent homology is then computed on the PCA-projected point cloud using a Vietoris–Rips filtration up to maxdim=1, yielding $H_{0}$ and $H_{1}$ persistence diagrams. In the default implementation, the diagram distance is the bottleneck distance:

(59) $$d_{topo}(\theta ,o,\alpha )=d_{B}\left(D_{\theta ,clean,0},D_{\theta ,o,\alpha }\right),$$

where $d_{B}(\cdot ,\cdot )=d_{B}^{\left(0\right)}+d_{B}^{\left(1\right)}$ is implemented as the sum of the bottleneck distances for $H_{0}$ and $H_{1}$. If the *ripser* or *persim* libraries are unavailable, the code falls back to a simple finite-dimensional surrogate; however, the intended manuscript-level runs assume the full persistent-homology pipeline.

For each encoder, a robust scale parameter is estimated from the empirical set of positive topology distances:

(60) $$\tau_{\theta }=median\{d_{topo}(\theta ,o,\alpha )>0\}.$$

The released implementation then defines the condition-wise normalized topology-based representation diagnostic index as

(61) $$R(\theta ,o,\alpha )=exp\left(-\frac{d_{topo}(\theta ,o,\alpha )}{\tau_{\theta }}\right).$$

To remain consistent with the exported CSV files, the implementation stores this condition-wise topology-based representation diagnostic quantity under the column name TSI. Conceptually, however, it corresponds to the normalized topology-based representation metric index R(θ,o,α), not yet to the stress-integrated summaries introduced in Section 4.6. Representative persistence diagrams and level-wise topology-based metric curves are generated as part of the topology analysis.

The resulting normalized topology-based representation diagnostic measure corresponds to the topology-based representation metric index defined in Section 3.

### 4.6. Integrated Metrics and Tri-Objective Summary

The tri-objective summary is computed by merging the stress-sweep table and the topology table on the keys (encoder,op,level). The clean condition is excluded from the integrated summaries, and only non-clean stress operators contribute to the final utility.

Let Δα=0.25 denote the level spacing of the stress grid. For each encoder, the implementation computes

(62) $${TSI}_{P}(\theta )=\sum_{\alpha }\bar{P}(\theta ,\alpha )\;\Delta \alpha ,$$

where $\bar{P}(\theta ,\alpha )$ is the mean accuracy across non-clean operators at level α. Likewise, the stress-integrated topology-based representation metric is

(63) $${TSI}_{R}(\theta )=\sum_{\alpha }\bar{R}(\theta ,\alpha )\;\Delta \alpha ,$$

where $\bar{R}$ is the mean normalized topology-based representation metric index across non-clean operators at level α. The integrated residual proxy safety burden is

(64) $$R_{safe}(\theta )=\sum_{\alpha }\bar{{sev}_{max}}(\theta ,\alpha )\;\Delta \alpha .$$

The scalar utility used in the implementation is

(65) $$U(\theta )=\lambda_{P}{TSI}_{P}(\theta )+\lambda_{R}{TSI}_{R}(\theta )-\lambda_{S}R_{safe}(\theta ),\;\mathrm{With}\;\lambda_{P}=\lambda_{R}=\lambda_{S}=1.$$

In addition to the integrated summaries, the implementation records per-encoder worst-case values,

$minP,\;max{sev}_{max},\;minR,$ and pairwise correlations among accuracy, severity, and topology-based representation metrics. The resulting normalized topology-based representation diagnostic measure corresponds to the topology-based representation metric index defined in Section 3.

Because the current implementation averages first over operators and then integrates over levels, the resulting summary should be interpreted as an operator-balanced stress integral rather than a probability-weighted expectation over real-world perturbation frequencies.

Detailed stress-integrated metric definitions and utility components are provided in Supplementary Table S3. To address statistical uncertainty in the single-run evaluation, we additionally computed confidence intervals from the existing test-set outputs. Accuracy confidence intervals were estimated using Wilson binomial confidence intervals based on the reported number of correct predictions and test-set size. For the tri-objective utility summaries, we used a condition-level percentile bootstrap over the non-clean encoder-level and operator-level rows. This bootstrap analysis was intended to quantify uncertainty in the reported single-run operating-envelope summaries, not to replace a full repeated-training or repeated-seed robustness analysis. In addition to the weighted utility summaries, we performed a simple Pareto-style dominance analysis across the three stress-integrated objective axes: TSI_P was treated as a maximization objective, TSI_R as a maximization objective, and R_safe as a minimization objective. An encoder was considered dominated if another encoder achieved equal or better values on all three axes and a strictly better value on at least one axis. This analysis was intended to complement, rather than replace, the weighted utility summaries.

### 4.7. Stochastic Fuzz-Based Stress Exploration

The released code implements a stochastic fuzzing procedure that complements the structured grid sweep. Unlike the placeholder multi-axis fuzzing design originally sketched in the manuscript, the actual implementation samples **one stress operator and one continuous stress level at a time**. Fuzzing is enabled in the manuscript-level final configuration and disabled in the pilot configuration.

For a given benchmark, fuzzing starts from a fixed seed set of test images, $N_{seed}=min(128,\;N_{test})$ in final mode. For each encoder, clean percepts and their corresponding latent representations are first computed on the seed set. A PCA model is then fitted on the clean latent representations and reused to embed mutated samples into a common low-dimensional representation space.

• At each fuzzing mutation, the procedure samples.
• One seed image index.
• One non-clean stress operator from {noise,blur,dropout,occlusion}.
• One continuous stress level α∼U(0,1).

The mutated image is encoded, projected to the feasible stimulation set, converted to a percept, and passed through the frozen benchmark-specific decoder. The code records the true label, predicted label, correctness indicator, residual proxy severity, and a representation-distance score

(66) $$d_{rep}={∥PCA(z)-PCA(z_{ref})∥}_{2},$$

where $z_{ref}$ is the first clean seed representation used as a fixed reference anchor in the current implementation.

Coverage is tracked by discretizing each mutated case into a tuple of the form $(\mathrm{encoder},\;b_{sev},\;b_{rep},\;\mathrm{correct}),$ where $b_{sev}=min(9,\lfloor 3\;sev\rfloor ),\;b_{rep}=min(9,\lfloor 2\;d_{rep}\rfloor )$.

A mutation contributes to a new coverage bin if the corresponding tuple has not previously been observed.

The code also assigns each candidate a scalar counterexample score

(67) $$s=sev+I(\hat{y}\neq y)+0.25\;d_{rep},$$

which assigns larger scores to cases with higher residual proxy safety burden, task failure, and representation displacement. In the final configuration, fuzzing uses 80 iterations×16 mutations per iteration for each encoder. Accordingly, the present fuzzing procedure should be understood as stochastic single-operator stress exploration rather than a full combinatorial optimizer over a multidimensional perturbation vector. The resulting fuzzing observations should therefore be interpreted as exploratory, benchmark-specific operating-region sampling within the evaluated EMNIST setting, rather than as a comprehensive deployment-level operating-envelope characterization or as a generalized characterization of realistic prosthetic-vision operating conditions.

## 5. Results

This section reports the empirical behavior of the proposed evaluation framework under structured stress conditions using the two-benchmark suite introduced in Section 4. Because the released implementation trains a benchmark-specific shared decoder within each benchmark and stores artifacts separately for *emnist_letters* and *coco_4cls*, all results are presented in a benchmark-aware manner. Specifically, each major result category is reported for both EMNIST Letters and the COCO-derived four-class benchmark, while cross-benchmark comparisons are reserved for the final tri-objective summary.

The results are organized as follows. Section 5.1 reports stress-induced performance degradation. Section 5.2 summarizes post-projection residual proxy safety-burden patterns. Section 5.3 presents exploratory topology-based representation metrics. Section 5.4 analyzes the coupling structure among accuracy, residual proxy severity, and topology-based representation metrics. Section 5.5 summarizes benchmark-specific and cross-benchmark tri-objective trade-offs. Section 5.6 reports stochastic fuzzing results and highlights candidate stress-sensitive operating regions not fully captured by the structured grid sweep. Section 5.7 summarizes the main empirical findings.

Because the COCO-derived benchmark contains four classes, the random-chance baseline is 0.25; therefore, relying solely on overall accuracy is insufficient for interpreting encoder-level tendencies in this benchmark. To address this issue, we report clean-condition diagnostic metrics including accuracy, balanced accuracy, macro-F1, the four-class chance baseline, and the accuracy-minus-chance margin in Table 2.

Table 2 shows that all four encoders operated only modestly above the four-class chance baseline, with accuracy margins ranging from 0.0500 to 0.0540. Thus, the COCO-derived benchmark should be interpreted as a low-performance, narrow-margin discrimination regime under the present simplified SPV percept-synthesis setting. Encoder-level differences in this benchmark should therefore be interpreted cautiously rather than as evidence of robust natural-scene understanding. To further characterize class-level behavior in the reduced four-class COCO-derived benchmark, Table 3 reports per-class precision, recall, F1-score, and support for each encoder under the clean condition. Because the test set was balanced across the four classes, each class had equal support of 250 samples.

The per-class results show that the compressed COCO-derived performance regime was not uniformly distributed across classes. Across encoders, the car class generally showed the highest recall, whereas the both class showed the lowest recall. This pattern indicates that the low overall accuracy was accompanied by class-specific weaknesses rather than by uniform degradation across the four categories. Therefore, the COCO-derived results should be interpreted as diagnostic evidence of a difficult, low-margin benchmark rather than as evidence of robust natural-scene recognition.

To complement the per-class diagnostic metrics, Table 4 reports the clean-condition confusion matrices for the reduced four-class COCO-derived benchmark. Rows indicate the true class labels, whereas columns indicate predicted class labels. The class order is none, person, car, and both.

The confusion matrices are consistent with the interpretation that the COCO-derived benchmark operated as a low-margin diagnostic regime. Across encoders, predictions were broadly distributed across the four classes rather than being concentrated along a strong diagonal. The both class was frequently confused with the single-object or none categories, consistent with the low recall values reported in Table 3. These results indicate that the COCO-derived benchmark should be interpreted as a difficult four-class diagnostic task under the present simplified SPV setting, rather than as evidence of reliable natural-scene recognition.

The COCO-derived diagnostic outputs corresponding to Table 2, Table 3 and Table 4 are provided in the supplementary output files. Specifically, Table 2 corresponds to Manuscript_Table_COCO_Diagnostic_Metrics.csv; Table 3 corresponds to COCO_classification_report_rate.csv, COCO_classification_report_sparse.csv, COCO_classification_report_temporal.csv, and COCO_classification_report_optim.csv; and Table 4 corresponds to COCO_confusion_matrix_rate.csv/png, COCO_confusion_matrix_sparse.csv/png, COCO_confusion_matrix_temporal.csv/png, and COCO_confusion_matrix_optim.csv/png. These files were regenerated from the same final fixed-seed canonical dataset used for the manuscript tables.

### 5.1. Stress-Induced Performance Degradation

**Figure 2:**
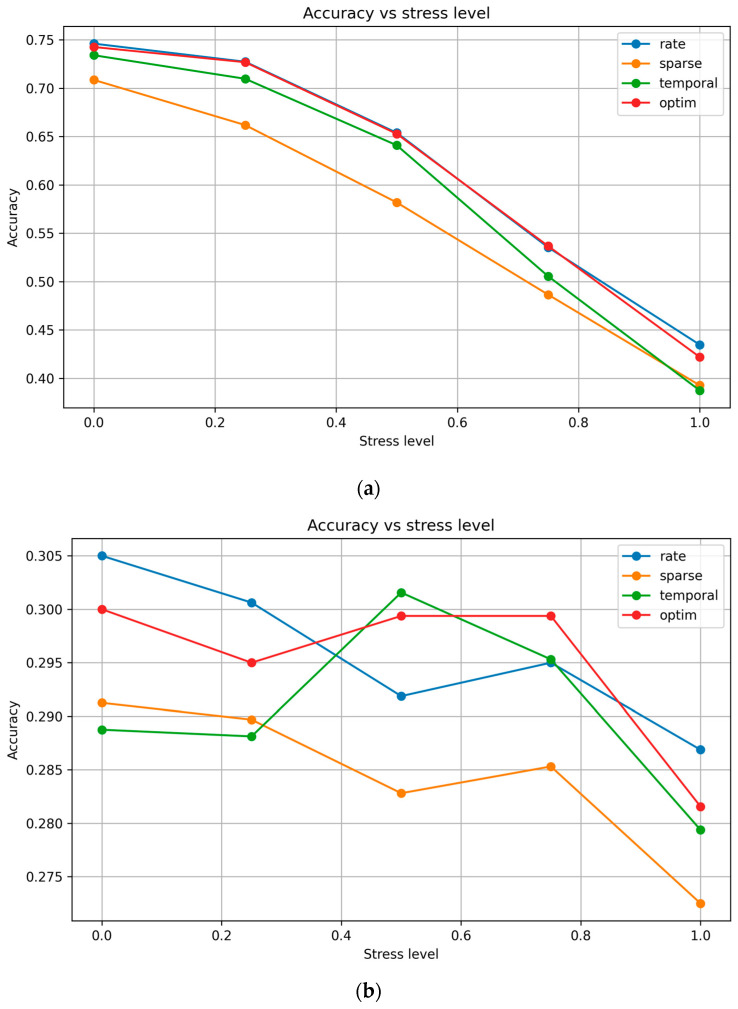
Accuracy versus stress level on (**a**) EMNIST Letters and (**b**) the COCO-derived four-class benchmark. Encoder-wise classification accuracy as a function of stress level for the symbolic-recognition benchmark in (**a**) and the reduced four-class COCO-derived benchmark in (**b**).

We first examined how task performance changed as the input stress level increased under the four implemented non-clean operators (noise, blur, dropout, and occlusion). Figure 2 summarizes the benchmark-specific accuracy trajectories for all compared encoders.

On the EMNIST benchmark, all encoders showed comparable clean-condition performance, with optim yielding the highest clean-condition accuracy (0.8020), followed closely by rate (0.8000) and temporal (0.7933), while sparse started from a slightly lower baseline (0.7730). However, the degradation trajectories under stress revealed clearer differences than the clean-condition ranking alone. Blur had only a limited effect on performance, whereas dropout, noise, and especially occlusion induced substantial degradation. Across all encoders, the worst observed accuracies occurred under severe occlusion, with minima of 0.1383 for rate, 0.1433 for sparse, 0.1297 for temporal, and 0.1437 for optim. These results indicate that, in the symbolic-recognition setting, the relative encoder ranking becomes increasingly condition-dependent as stress severity increases.

Clean-condition performance was uniformly lower in the COCO-derived benchmark than in EMNIST, with diagnostic clean-condition accuracy values ranging from 0.3000 to 0.3040 across encoders, indicating that the reduced four-class COCO-derived task remained a low-margin discrimination problem under the present simplified SPV percept representation. At the same time, the stress-induced degradation curves were markedly flatter than those observed on EMNIST. Across operators and levels, accuracy remained in a relatively narrow band, and worst-case accuracies were 0.2890 for rate, 0.2670 for sparse, 0.2780 for temporal, and 0.2590 for optim.

In this benchmark, the most adverse cases arose under occlusion, noise, or dropout depending on the encoder, but the absolute performance drop from the clean condition was modest compared with EMNIST. Thus, unlike the symbolic-recognition task, the reduced COCO-derived benchmark did not show a large dynamic range of degradation; rather, it operated in a compressed low-accuracy regime in which differences among encoders were more subtle at the level of accuracy alone. Nevertheless, severe stress conditions still produced downward trends across encoder configurations, indicating that the framework remained sensitive to progressive degradation even under compressed performance conditions.

Table 5 shows that clean-condition task accuracy and residual proxy safety burden were not uniformly aligned across encoder configurations. In the EMNIST benchmark, the temporal encoder achieved competitive clean-condition accuracy but also produced substantially higher residual proxy safety-burden metrics than the sparse encoder, which maintained near-zero residual severity at the cost of a modest reduction in baseline accuracy. Similar tendencies were observed in the reduced COCO-derived benchmark, although the overall accuracy range remained substantially compressed across encoder types. These results indicate that encoder evaluation under phosphene-constrained conditions cannot be reduced to task accuracy alone, since encoder configurations with comparable clean-condition accuracy may nevertheless exhibit substantially different residual stimulation characteristics. Table 6 further highlights that the stress operator producing the worst accuracy was not necessarily identical to the operator producing the highest mean residual proxy safety burden. Occlusion consistently produced the lowest task accuracy across most encoder configurations, whereas blur or clean-condition temporal stimulation often yielded the highest mean residual proxy safety-burden metrics depending on the encoder type. This discrepancy indicates that task-level degradation and stimulation-related burden do not always evolve monotonically under identical stress conditions. Consequently, the framework separates functional degradation from residual stimulation burden rather than treating them as interchangeable indicators of stress response. Taken together, Figure 2 and Table 5 and Table 6 show that nominal clean-condition performance was only partially predictive of stressed behavior. In EMNIST, stress revealed substantial operator-dependent divergence among encoders, particularly under noise, dropout, and occlusion.

In contrast, the COCO-derived benchmark was characterized by a lower but more compressed accuracy regime, suggesting that benchmark difficulty manifested less as steep degradation from a high baseline and more as persistently limited task separability under phosphene-constrained viewing.

### 5.2. Post-Projection Residual Proxy Safety-Burden Patterns

**Figure 3:**
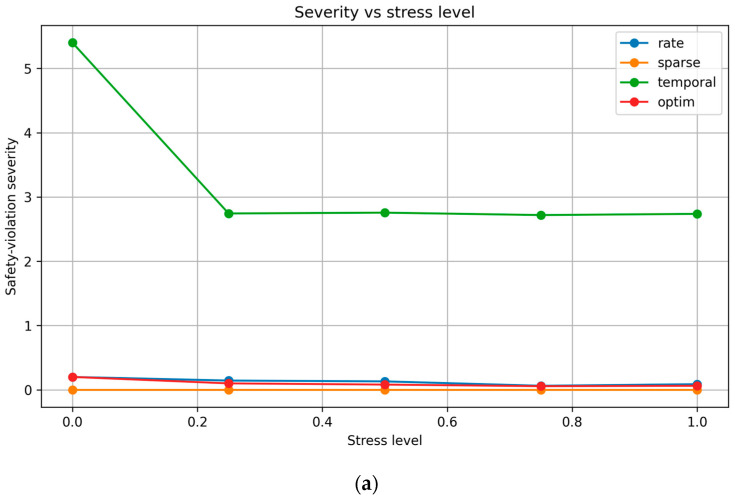

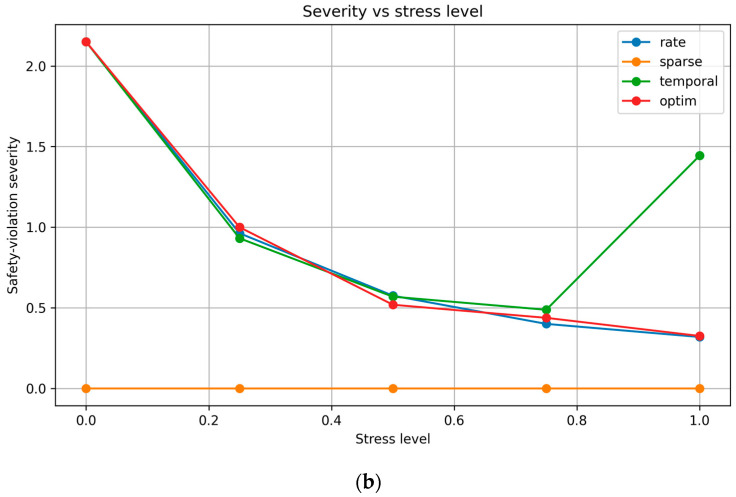
Post-projection residual proxy severity versus stress level on (**a**) EMNIST Letters and (**b**) the COCO-derived benchmark. Encoder-wise maximum post-projection residual proxy severity under the structured stress sweep for the symbolic-recognition benchmark in (**a**) and the reduced COCO-derived benchmark in (**b**).

Figure 3 shows benchmark- and encoder-dependent variation in residual post-projection proxy severity across the evaluated stress levels.

The post-projection residual severity trajectories revealed a markedly different structure from the accuracy curves. On EMNIST, the sparse encoder maintained an almost negligible residual proxy safety burden throughout the structured stress sweep, with a clean-condition maximum severity of only $2.26\times 10^{-7}$ and a worst-case maximum severity of $2.88\times 10^{-7}$. By contrast, the temporal encoder exhibited the largest residual proxy safety burden under the evaluated stress configuration, with a clean-condition maximum severity of 5.4, which also remained its worst observed severity over the sweep. The rate and optim encoders occupied an intermediate regime: both showed modest but non-negligible clean-condition severity (0.2), and their worst residual burden increased only slightly to 0.225, occurring under blur at level 0.5. Thus, in the symbolic-recognition benchmark, the encoder ranking by residual proxy safety burden was highly asymmetric, with sparse more distinctly separated from temporal and the remaining two encoders clustered in between. The strong encoder dependence observed in the residual proxy safety-burden analysis can be interpreted in relation to the temporal and spatial concentration characteristics of the evaluated stimulation protocols. In particular, the temporal encoder frequently generated concentrated activation bursts that accumulated disproportionately large duty-related and proxy-charge feasibility violations under severe stress conditions. By contrast, the sparse encoder naturally distributed activation across fewer phosphene channels and therefore remained substantially below several feasibility thresholds throughout most operating conditions. These observations suggest that encoder-specific stimulation geometry plays a major role in determining residual feasibility burden independently of decoder-level task performance.

The COCO-derived benchmark showed a stronger and more widespread residual burden. Sparse again remained effectively at zero severity, with a clean-condition maximum severity of $2.26\times 10^{-7}$ and a worst-case value of $2.88\times 10^{-7}$. However, the other three encoders all began from a substantially elevated clean-condition severity of 0.5000, indicating that the reduced COCO-derived task placed the system closer to the feasible-operating boundary even before additional stress was introduced. Among them, temporal again showed the largest residual proxy safety burden, reaching a worst-case maximum severity of 5.4 under occlusion at level 1.0. Rate and optim retained the same worst-case maximum severity as their clean-condition value, indicating that for these encoders the largest residual proxy burden was already present at the nominal operating point rather than being induced primarily by progressive external stress.

These results should be interpreted jointly with the accuracy trajectories in Section 5.1. In EMNIST, encoder ranking by accuracy and encoder ranking by residual severity were only partially aligned: sparse began from the lowest clean accuracy among the four encoders, yet showed by far the lowest residual proxy-burden profile whereas temporal retained competitive clean performance but incurred an exceptionally large residual proxy safety burden. The same dissociation became even more pronounced on the COCO-derived benchmark, where accuracy differences among encoders were relatively compressed while differences in residual proxy safety burden remained large. In other words, performance alone would have suggested only modest separation among several encoders, whereas the residual proxy safety-burden analysis exposed a much clearer distinction in feasible operating behavior.

Because the implementation also stores the mean severity and violation rate, the present results indicate not only isolated high-severity cases but persistent differences in operating regime. On EMNIST, sparse combined the lowest maximum severity with the lowest mean severity, while temporal combined the largest maximum severity with the largest mean severity. On the COCO-derived benchmark, sparse again remained uniquely favorable, whereas rate, temporal, and optim all showed high clean-condition violation levels and nontrivial average residual-burden. Therefore, the proposed framework revealed benchmark-dependent differences in the relationship between task performance and residual proxy safety burden. In the reduced COCO-derived benchmark, the compressed low-accuracy regime produced weaker alignment between performance and residual proxy safety-burden metrics under stress conditions. This supports the more limited interpretation that accuracy-only evaluation may mask differences in how the evaluated encoder–decoder configurations accumulate residual post-projection proxy burden under stress.

### 5.3. Exploratory Topology-Based Representation Metrics

**Figure 4:**
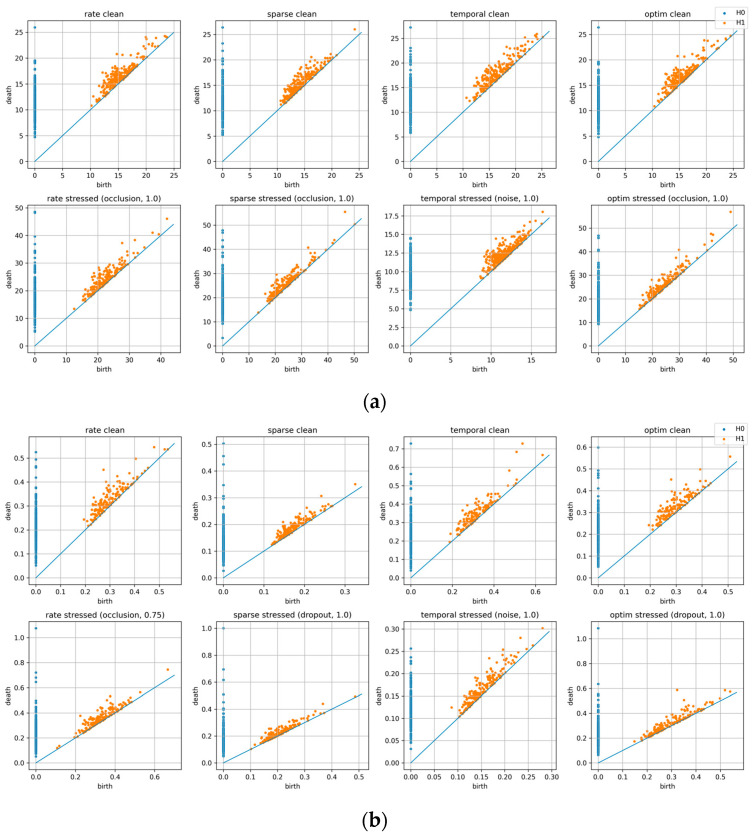
Representative persistence diagrams for (**a**) EMNIST Letters and (**b**) the reduced COCO-derived benchmark under clean and maximally displaced stress conditions. Each point represents a topological feature characterized by its birth and death coordinates during filtration, where points farther from the diagonal correspond to more persistent topological structures. Clean-condition diagrams are compared with stress condition exhibiting the largest topology-based displacement for each encoder configuration in order to visualize stress-induced changes in representation-level organization.

Figure 4 shows representative persistence diagrams for the clean and most stressed conditions on the two benchmarks, highlighting the structural changes in the representation space under severe stress. The persistence diagrams in Figure 4 provide a qualitative visualization of how the topological organization of the induced representation space changes under stress. In these diagrams, points located farther from the diagonal correspond to more persistent topological structures, whereas points concentrated near the diagonal indicate weaker or shorter-lived topological features. Relative to the clean-condition diagrams, the stressed conditions generally exhibited compressed point distributions and altered clustering structure, indicating that severe stress modifies the representation-level topology produced by the encoder outputs. The magnitude and qualitative form of these changes differed across encoder configurations and benchmark regimes, consistent with the topology-based representation metric trends quantified in the subsequent analyses.

**Figure 5:**
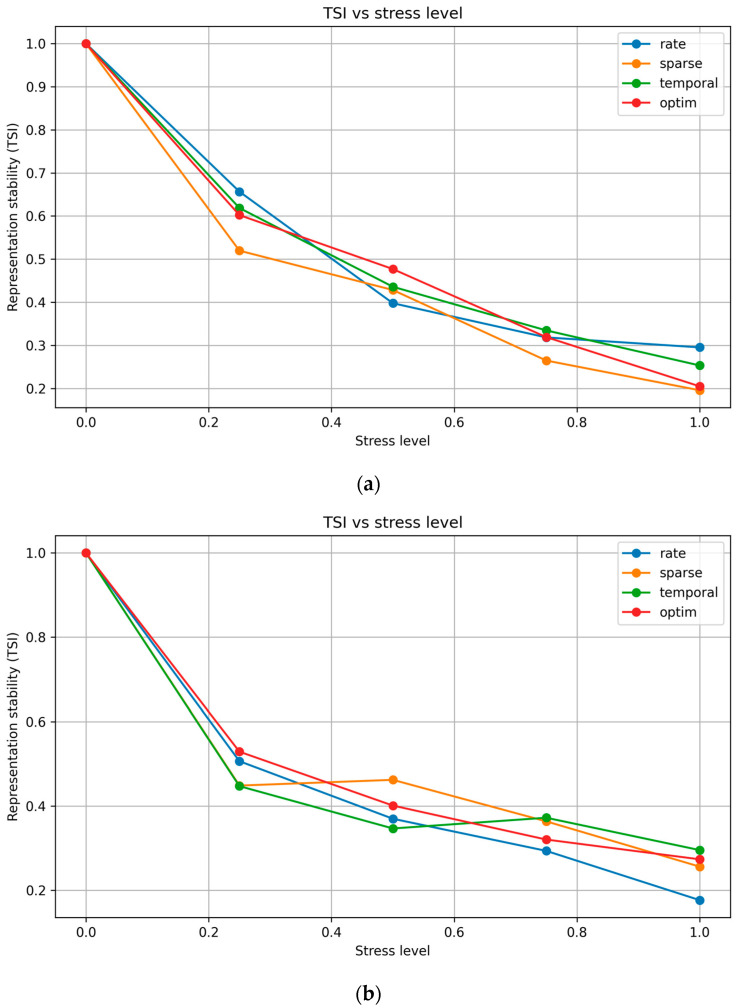
The topology-based representation metric versus stress level on (**a**) EMNIST Letters and (**b**) the COCO-derived benchmark. Encoder-wise mean condition-wise topology-based representation index across stress levels for the symbolic-recognition benchmark in (**a**) and the reduced COCO-derived benchmark in (**b**).

We next turn to the exploratory topology-based representation metric. Figure 5 shows the topology-based representation index as a function of stress level for the two benchmarks. This quantity should be interpreted as an exploratory topology-based representation diagnostic derived from persistent-homology-based deviation from the clean condition, rather than as a validated general stability measure. For each encoder and benchmark, the released implementation computes decoder-latent embeddings, fits PCA on the clean condition, projects stressed latent clouds into the same subspace, and measures topological deviation using persistent homology. The resulting normalized topology-based representation index is stored in the benchmark-specific topology table under the column TSI, corresponding to the exploratory topology-based metric defined in Equation (25).

Detailed benchmark-specific topology metrics are provided in Supplementary Tables S4 and S5.

On EMNIST, all encoders began with the clean-condition reference value TSI = 1, but topology-based representation index declined steadily as stress severity increased. When averaged across non-clean operators and levels, the rate encoder showed the highest mean topology-based representation index (0.5335), followed closely by temporal (0.5284) and optim (0.5205), whereas sparse exhibited the lowest mean topology-based representation index (0.4816). Across operators, blur was the least disruptive condition, with the highest mean TSI and the smallest topological displacement, while noise produced the strongest average degradation, followed by occlusion. In particular, sparse was especially sensitive to noise at the representation level, yielding the lowest mean TSI among the four encoders under that operator. Thus, in the symbolic-recognition benchmark, topology-based metric differentiated encoder behavior even when clean-condition performance appeared relatively similar.

The COCO-derived benchmark showed a different but equally informative pattern. As in EMNIST, all encoders had TSI = 1 in the clean condition, but stress rapidly reduced topology-based representation metrics even though task accuracy itself remained in a relatively compressed range. When averaged across non-clean operators and levels, sparse (0.5058) and optim (0.5045) showed the highest mean TSI, followed by temporal (0.4921), while rate yielded the lowest mean topology-based representation index (0.4690). Compared with the EMNIST benchmark, the reduced COCO-derived benchmark exhibited a more compressed topology-metric range across encoder configurations and stress levels. Nevertheless, the TSI trajectories continued to decrease progressively with increasing stress, indicating that the topology-based representation metric remained sensitive to stress-induced structural variation even under comparatively compressed decoder-accuracy conditions. Encoder-specific differences were still observable, although the separation between curves became less pronounced than in the symbolic-recognition benchmark.

Noise again produced the strongest average topological disruption, whereas blur remained the least disruptive operator. Importantly, at high stress levels the TSI values fell substantially for all encoders, often into the range of roughly 0.18–0.30, despite only modest changes in classification accuracy relative to the clean baseline. This indicates that, in the reduced COCO-derived benchmark, topology-based representation metrics exhibited larger relative variation than decoder accuracy under the evaluated stress conditions.

This contrast between the two benchmarks is central. In EMNIST, the decline in topology-based representation metric largely tracked the decline in task accuracy: as stress increased, both performance and representation structure deteriorated together. By contrast, in the reduced COCO-derived benchmark, the topology curves exhibited a broader dynamic range than the corresponding accuracy curves. Across several encoder–stress combinations, changes in task performance remained relatively limited, whereas topology-based representation metrics varied more substantially. These observations suggest weaker coupling between decoder accuracy and topology-based representation metrics under degraded conditions in the reduced COCO-derived benchmark. In this sense, topology-based analysis provided complementary structural information regarding representation degradation that was not fully reflected in decoder accuracy alone.

Taken together, Figure 5 and Supplementary Tables S4 and S5 show that the topology-based representation metrics provide information that is not reducible to output-level performance. In EMNIST, it offers a structurally consistent companion measure to the degradation curves in Section 5.1. In the COCO-derived benchmark, however, topology-based analysis provided complementary structural information that was not fully reflected in accuracy alone. These findings motivate the subsequent coupling analysis in Section 5.4, where the relationship among accuracy, residual proxy safety burden, and topology-based representation metric is examined more explicitly.

### 5.4. Accuracy, Residual Severity, and Exploratory Topology-Based Representation Metrics

**Figure 6:**
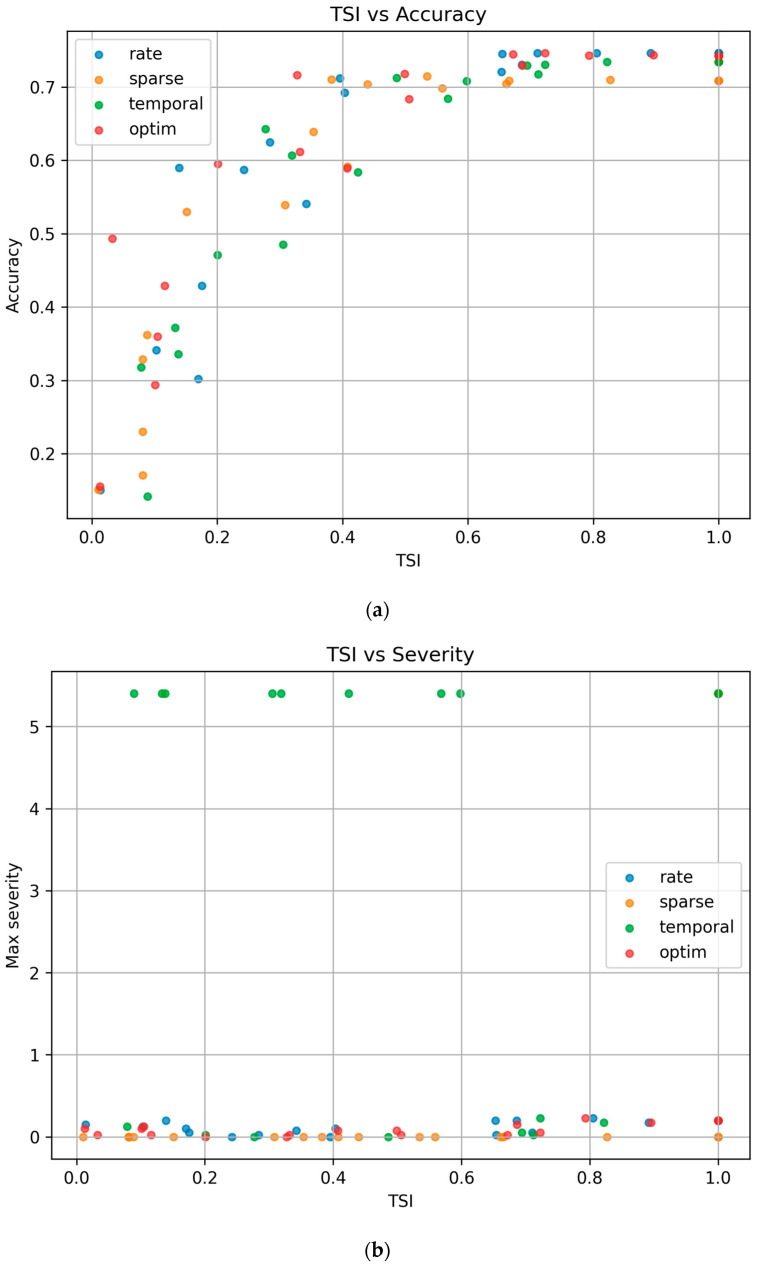
Topology-based representation metric versus accuracy and residual severity on EMNIST Letters. Condition-wise coupling between the normalized topology-based representation metric index and accuracy, and between the normalized topology-based representation metric index and residual proxy severity for the symbolic-recognition benchmark.

**Figure 7:**
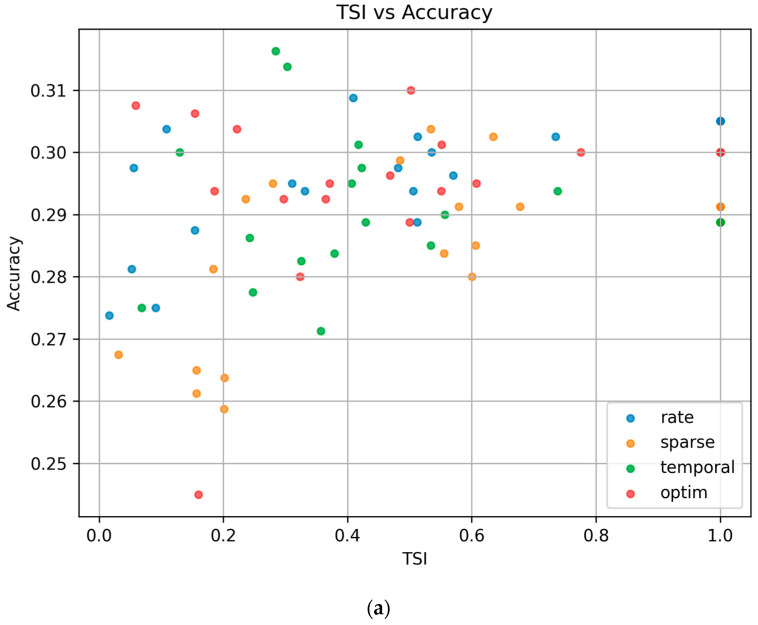

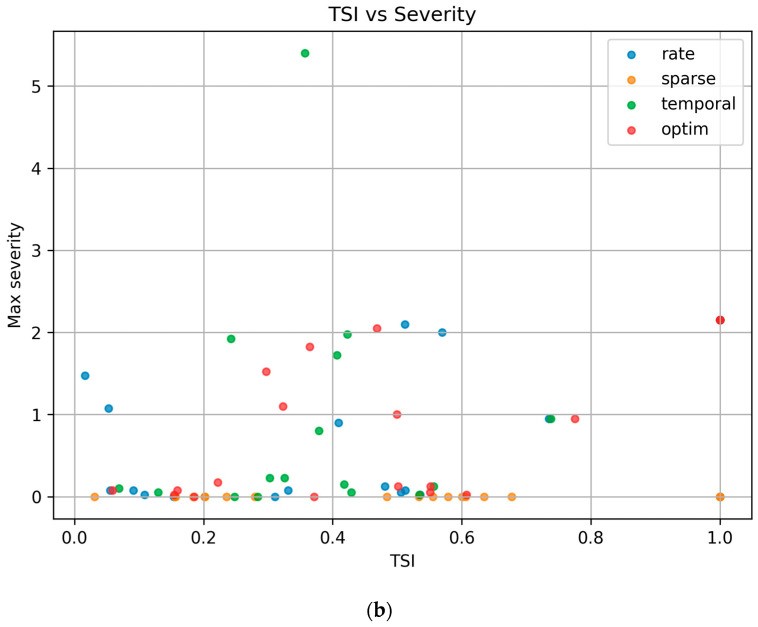
Topology-based representation metric versus accuracy and residual severity on the COCO-derived benchmark. Condition-wise coupling between the normalized topology-based representation metric index and accuracy, and between the normalized topology-based representation metric index and residual proxy severity for the reduced COCO-derived four-class benchmark.

We next analyze how the three axes of the proposed evaluation framework interact. The benchmark-specific correlation statistics are summarized in Table 7, and two coupling plots in Figure 6 and Figure 7 are presented for each benchmark: topology-based representation metric versus accuracy and topology-based representation metric versus residual severity.

Figure 6a shows that the topology-based representation metric and decoder accuracy generally exhibited positive coupling across the evaluated stress conditions. As stress increased, most encoder-conditioning pairs shifted toward the lower-left region of the plot, indicating simultaneous reduction in topology-based representation metrics and task performance. However, the clustering structure differed across encoder configurations, suggesting that the rate and trajectory of degradation were encoder dependent rather than globally uniform.

By contrast, the relationship between topology-based representation metric and residual proxy safety burden in Figure 6b was substantially more asymmetric. In particular, the temporal encoder maintained consistently elevated residual severity across a broad range of TSI values, whereas the sparse encoder remained concentrated near negligible severity despite topology degradation under stress. These observations indicate that topology-based representation variation and residual stimulation burden do not evolve monotonically together and therefore provide complementary rather than interchangeable diagnostic information.

Compared with the EMNIST benchmark, the reduced COCO-derived benchmark exhibited substantially weaker coupling between the topology-based representation metric and decoder accuracy. In Figure 7a, the accuracy values remained confined to a comparatively compressed range despite substantial variation in TSI across stress conditions and encoder configurations. This indicates that representation-level topology metrics continue to vary under stress even when decoder-accuracy differences became less strongly separated.

The relationship between topology-based representation metric and residual proxy safety burden in Figure 7b also remained highly asymmetric across encoder configurations. In particular, the temporal encoder produced several high-severity outliers across a broad TSI range, whereas the sparse encoder remained concentrated near negligible residual severity despite progressive topology degradation. These observations further support the interpretation that topology-based representation metric, task accuracy, and residual stimulation burden capture complementary aspects of the stress response under phosphene-constrained conditions.

Benchmark-specific correlation summaries are reported in Table 7. The coupling analysis showed that the topology-based representation metric was generally more closely aligned with task performance than with residual severity, but the strength of this alignment was benchmark-dependent. On EMNIST, all encoders exhibited a strong positive correlation between TSI and accuracy, with corr(TSI, Acc) ranging from 0.8002 to 0.8421. This indicates that, in the symbolic-recognition benchmark, deterioration of representation structure was closely mirrored by output-level performance loss. The strongest such coupling was observed for the temporal encoder (0.8421), followed by optim (0.8198), rate (0.8069), and sparse (0.8002). In other words, for EMNIST the topology-based metric captured representation-level variation associated with stressed functional performance.

The relationship between topology-based representation metric and residual severity was more heterogeneous. On EMNIST, rate and optim showed moderate positive corr(TSI, Sev) values of 0.5127 and 0.6920, respectively, whereas sparse and temporal showed only weak associations, with corr(TSI, Sev) values near 0.08. Thus, in the symbolic-recognition benchmark, severity was not uniformly coupled to topology-based representation metrics across encoders. Instead, some encoders showed gradual joint changes in topology-based representation metrics and residual proxy safety burden, whereas others exhibited largely decoupled behavior. This asymmetry indicates that residual proxy safety burden cannot be inferred from representation change alone.

The COCO-derived benchmark yielded a qualitatively different structure. Although sparse and rate still showed positive corr(TSI, Acc) values of 0.6022 and 0.6776, respectively, the relationship weakened substantially for optim (0.2590) and nearly disappeared for temporal (−0.0356). Hence, in the reduced COCO-derived benchmark, the link between topology-based representation metric and task performance became much less uniform. This result is consistent with the interpretation advanced in Section 5.3: under COCO-like conditions, topology-based representation metrics sometimes varied substantially without correspondingly large changes in decoder accuracy, especially for the temporal encoder. In this regime, topology-based representation metric was not merely a surrogate for performance, but a complementary diagnostic axis that provided additional representation-level variation information beyond output-level accuracy trends.

The coupling between topology-based representation metrics and residual severity was also benchmark-dependent. In COCO, optim and rate showed relatively strong positive corr(TSI, Sev) values of 0.6658 and 0.6453, respectively, whereas sparse remained weakly coupled (0.1290) and temporal showed only moderate association (0.3732). At the same time, the direct correlation between accuracy and severity remained weak across all encoders in both benchmarks, with corr(Acc, Sev) values never exceeding 0.2570 in EMNIST and 0.1851 in COCO, and with temporal even showing negative corr(Acc, Sev) in both datasets. Therefore, the coupling results indicate that performance and residual proxy safety burden were only loosely associated within the evaluated conditions, and that the topology-based representation metric occupied an intermediate diagnostic role: in some settings it tracked performance closely, whereas in others it revealed representation-level variation that was only weakly reflected in either performance or residual severity.

Taken together, these benchmark-specific coupling patterns support two conclusions. First, topology-based representation metric behaves as a useful companion diagnostic to performance in EMNIST, where degradation of latent structure and degradation of output accuracy remain tightly linked. Second, in the COCO-derived benchmark the coupling becomes weaker and more encoder-dependent, indicating that topology-based representation metric provides additional information beyond both accuracy and residual severity. This benchmark asymmetry strengthens the overall claim of the paper that single-metric evaluation is insufficient for characterizing phosphene-encoding behavior under stress.

### 5.5. Tri-Objective Trade-Off Structure

**Figure 8:**
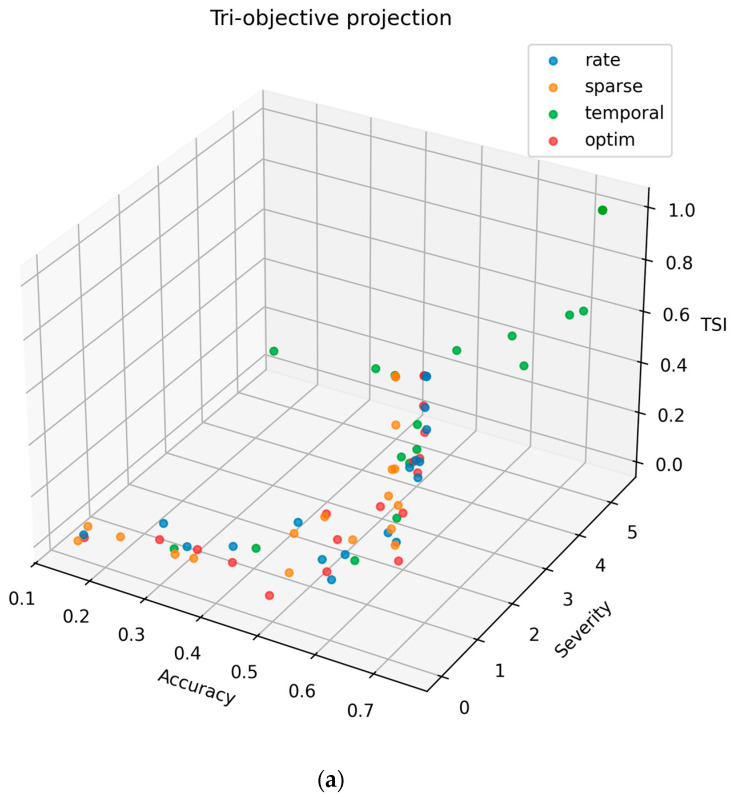

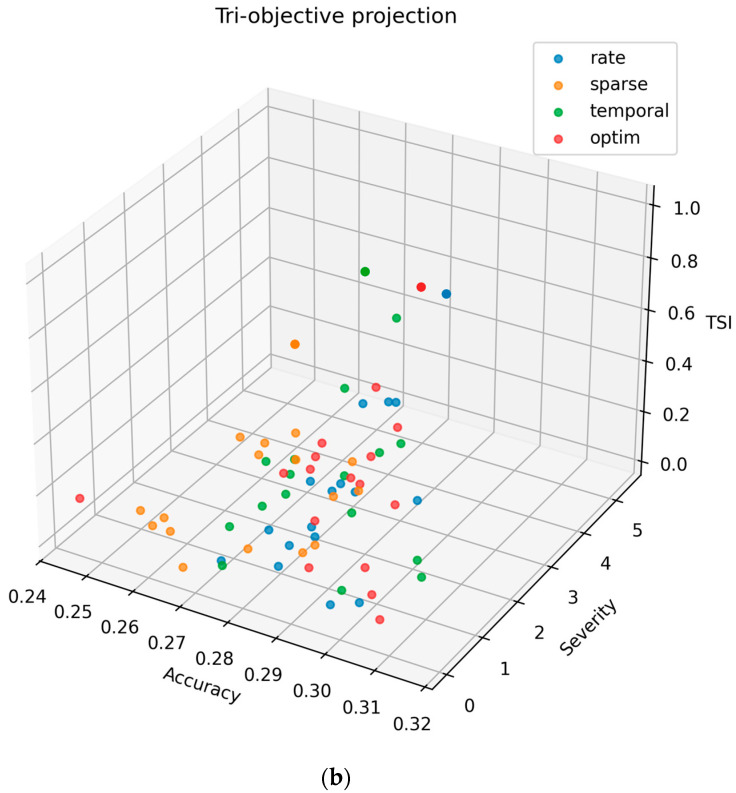
Tri-objective projection on EMNIST Letters and the COCO-derived benchmark. Condition-wise operating-envelope geometry in the (P, sev, R) space for (**a**) the symbolic-recognition benchmark and (**b**) the reduced COCO-derived benchmark.

We now summarize the full tri-objective operating envelope. For each benchmark, the released implementation aggregates stress-sweep accuracy, topology-based representation metric, and residual severity into stress-integrated summaries and computes an equal-weight utility score. Benchmark-specific tri-objective projections are shown in Figure 8.

Figure 8 visualizes the benchmark-specific operating-envelope geometry in the joint space of task accuracy, residual proxy safety burden, and the topology-based representation metric. In this tri-objective space, favorable operating regions correspond to simultaneously high accuracy, low residual severity, and high topology-based representation metrics, whereas stress-induced degradation shifts operating points toward lower TSI and increased residual burden. The evaluated encoder–decoder configurations formed distinct operating clusters across the two benchmarks, indicating that they occupied different trade-off regimes rather than converging to a single common trade-off pattern. In particular, the sparse encoder generally remained concentrated in low-severity regions, whereas the temporal encoder occupied operating regions associated with substantially elevated residual proxy safety burden despite competitive task performance under several conditions.

A benchmark-integrated summary table is given in Table 8. The table was regenerated from the final fixed-seed canonical dataset and reports the deterministic stress-integrated point-estimate utility values that are subsequently uncertainty-qualified using the bootstrap confidence intervals in Table 9.

The deterministic point-estimate utility in Table 8 and the bootstrap mean utility in Table 9 are aligned because both were computed from the same final fixed-seed canonical stress-integrated outputs under the baseline equal-weight evaluation policy. The bootstrap confidence intervals quantify condition-level uncertainty over the non-clean encoder-level and operator-level rows and should therefore be interpreted as uncertainty summaries for the observed single-run operating envelope, not as repeated-training or repeated-seed statistical inference. On EMNIST Letters, the confidence intervals for rate, sparse, and optim overlapped substantially, indicating that the corresponding utility ranking should be interpreted as a descriptive single-run ordering rather than as statistically conclusive superiority. On the reduced COCO-derived benchmark, sparse showed the highest point-estimate utility and a confidence interval separated from those of the other encoders under the present condition-level bootstrap analysis. Even in this case, the result remains specific to the simplified SPV percept-synthesis setting, the baseline utility definition, and the fixed benchmark-specific decoder.

Importantly, the favorable point-estimate utility of the sparse encoder did not arise from the highest stress-integrated performance alone. In both benchmarks, sparse did not maximize TSI_P; rather, its utility advantage was primarily associated with a near-zero integrated residual proxy safety burden together with competitive topology-based representation metrics. On EMNIST, this advantage should be regarded cautiously because the bootstrap confidence intervals overlapped with those of rate and optim. On the COCO-derived benchmark, sparse remained more clearly separated under the current condition-level bootstrap analysis; however, this encoder preference should be read within the benchmark’s low-performance, narrow-margin four-class discrimination regime and remains specific to the simplified SPV percept-synthesis operator, the selected utility definition, and the fixed benchmark-specific decoder. Accordingly, this observation should be interpreted as a benchmark-specific and configuration-dependent joint encoder–decoder stress response under the present fixed-observer protocol, not as an encoder-only robustness claim, broad natural-scene robustness, or deployment-level characterization. It should not be generalized to physiologically calibrated phosphene generation, subject-specific phosphene maps, implant-aware stimulation constraints, or temporal percept dynamics. To examine whether the tri-objective utility ranking depended on the assumed operating preference, we recomputed the post hoc utility under four weighting scenarios: balanced $\left(\lambda_{P},\lambda_{R},\lambda_{S})=(1/3,1/3,1/3\right)$, performance-dominant (0.6, 0.2, 0.2), topology-metric-dominant (0.2, 0.6, 0.2), and proxy-burden-dominant (0.2, 0.2, 0.6). In this sensitivity analysis, the decision axes were expressed as P = TSI_P, R = TSI_R, and S = 1 − R_safe so that larger values consistently indicated more favorable operating behavior across all three axes. Therefore, the values in Table 10 should be interpreted as post hoc preference-sensitivity scores rather than as direct numerical replacements for the deterministic point-estimate utility values in Table 8.

The weight-sensitivity analysis showed that the utility-based encoder ordering was preference-dependent in EMNIST Letters but more stable in the reduced COCO-derived benchmark. On EMNIST Letters, sparse yielded the highest post hoc utility under the balanced, topology-metric-dominant, and proxy-burden-dominant scenarios, whereas the performance-dominant scenario shifted the highest utility to rate, with optim remaining close. This indicates that the apparent advantage of sparse in EMNIST depends strongly on how much residual proxy safety-burden control is weighted relative to stress-integrated performance. On the reduced COCO-derived benchmark, sparse yielded the highest post hoc utility across all four weighting scenarios, primarily because its near-zero integrated residual proxy safety burden consistently improved its decision score. However, this result should still be interpreted as a preference-sensitivity result within the present fixed-seed, simplified SPV, and fixed-decoder configuration, not as a statistically definitive or physiologically general encoder superiority claim. These results indicate that the proposed utility should be treated as a preference-dependent decision aid rather than as an absolute encoder hierarchy. To further assess the multi-objective structure independently of a scalar weighting policy, we additionally performed a Pareto-front membership analysis across TSI_P, TSI_R, and R_safe.

**Figure 9:**
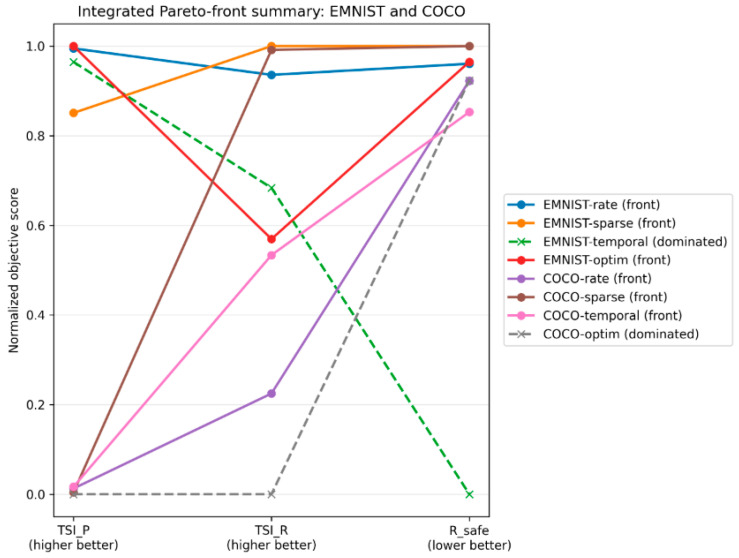
Integrated Pareto-front summary across EMNIST Letters and the reduced COCO-derived benchmark. The three stress-integrated objective axes are shown in a normalized parallel-coordinate format: TSI_P and TSI_R were treated as maximization objectives, whereas R_safe was inverted after normalization so that higher values indicate a more favorable objective value on all three axes. Solid circle-marked lines denote non-dominated encoder configurations, whereas dashed x-marked lines denote dominated configurations. Normalization was used only for visualization; Pareto-front membership was computed from the original unnormalized objective values reported in Table 11.

Figure 9 visualizes the Pareto-front structure in a normalized parallel-coordinate format in order to make the three stress-integrated objectives directly comparable. Because TSI_P and TSI_R are favorable when larger, they are displayed in the upward direction. By contrast, R_safe is favorable when smaller; therefore, it was inverted after min–max normalization so that higher plotted values consistently indicate more favorable operating behavior across all three axes. Importantly, this normalization was used only for visualization; Pareto-front membership and dominance relationships were computed from the original unnormalized objective values reported in Table 11. The visualization shows that the sparse encoder occupied a favorable low-burden region in both benchmarks because of its near-zero integrated residual proxy safety burden, even though it did not always maximize stress-integrated performance. On EMNIST Letters, rate, sparse, and optim remained non-dominated, whereas temporal was dominated by rate due to its substantially larger residual proxy safety burden. On the reduced COCO-derived benchmark, rate, sparse, and temporal were non-dominated, whereas optim was dominated by sparse. Thus, the Pareto-front visualization reinforces that encoder preference is trade-off-dependent and cannot be reduced to a single absolute ranking.

The Pareto-front membership analysis showed that the non-dominated set differed across benchmarks. On EMNIST Letters, rate, sparse, and optim formed the Pareto front, whereas temporal was dominated by rate because it combined lower stress-integrated performance and topology-based representation metrics with a substantially larger integrated residual proxy safety burden. On the reduced COCO-derived benchmark, rate, sparse, and temporal were non-dominated, whereas optim was dominated by sparse. These results indicate that sparse should not be interpreted as strictly Pareto-dominant over all alternatives. Rather, sparse occupied a favorable proxy-burden-controlled region because of its near-zero integrated residual proxy safety burden, while other encoders retained competitive stress-integrated performance or topology-based representation metrics under specific benchmark conditions. Thus, the Pareto-front analysis supports the interpretation that encoder preference is multi-objective and trade-off-dependent rather than reducible to a single absolute hierarchy. The Pareto-front figure, Table 11, manuscript interpretation, and supplementary Pareto output file were regenerated from the same final fixed-seed canonical dataset to maintain consistency across the manuscript and response materials.

A second notable finding is that the reduced COCO-derived benchmark imposed a distinct tri-objective regime from the symbolic-recognition benchmark. In EMNIST Letters, the Pareto-front set included rate, sparse, and optim, indicating that no single encoder dominated all others across stress-integrated performance, topology-based representation metrics, and residual proxy safety burden. In the reduced COCO-derived benchmark, rate, sparse, and temporal were non-dominated, whereas optim was dominated by sparse. This result indicates that benchmark difficulty did not translate into a single uniform encoder hierarchy. Rather, different encoders occupied different regions of the trade-off space depending on the balance among TSI_P, TSI_R, and R_safe.

Taken together, these benchmark-specific and cross-benchmark summaries show that the proposed tri-objective framework does more than rank encoders by aggregate performance. It reveals which encoders remain favorable because they control residual proxy safety burden, which encoders retain competitive stress-integrated performance or topology-based representation metrics, and how the overall operating-envelope geometry shifts with task type. In the present experiments, sparse tended to occupy a favorable observed trade-off region across both symbolic and reduced COCO-derived tasks within the simplified SPV percept-synthesis and fixed-decoder evaluation setting, primarily because of its near-zero integrated residual proxy safety burden. However, the Pareto-front analysis also shows that sparse should not be interpreted as a universally dominant encoder or as evidence of encoder-only robustness. These analyses should be interpreted as complementary decision summaries rather than interchangeable statistical tests. Table 8 reports deterministic stress-integrated point-estimate utility values under the baseline equal-weight policy. Table 9 adds condition-level bootstrap confidence intervals to qualify the uncertainty of those single-run utility summaries. Figure 9 and Table 11 evaluate Pareto-front membership independently of a specific scalar weighting policy, whereas Table 10 evaluates how the scalar utility changes under alternative operating-preference weights. Therefore, agreement among these summaries provides descriptive support for a favorable operating region, but it should not be interpreted as statistically definitive encoder superiority. Overall, encoder preference in the present study should be interpreted as uncertainty-qualified, preference-dependent, and configuration-dependent within the simplified SPV percept-synthesis and fixed-decoder evaluation setting.

The supplementary output files corresponding to the tri-objective analyses in Section 5.5 are explicitly mapped to the manuscript tables and figure for reproducibility checking. Table 8 corresponds to CrossBenchmark_TriObjective_Summary.csv; Table 9 corresponds to Manuscript_Table_Utility_ConditionBootstrap_CI.csv; Table 10 corresponds to Manuscript_Table_Utility_WeightSensitivity.csv; Figure 9 corresponds to Manuscript_Table_Pareto_Frontier.csv; and Table 11 corresponds to Manuscript_Table_Pareto_Dominance.csv. These supplementary outputs were regenerated from the same final fixed-seed canonical dataset used for the revised manuscript tables, figures, and response materials.

### 5.6. Stochastic Fuzzing Results and Exploratory Operating-Region Sensitivity Patterns

The final analysis complements the structured grid sweep with stochastic fuzzing. Unlike the earlier placeholder design based on a multi-axis perturbation vector, the released implementation samples one non-clean stress operator and one continuous stress level at a time and ranks mutated cases using a composite score that combines residual severity, task failure, and representation displacement. In the current execution, benchmark-specific fuzzing artifacts were generated for EMNIST Letters only; therefore, the fuzzing analysis below is restricted to the symbolic-recognition benchmark and to the implemented single-operator stochastic stress configuration. Accordingly, these results should be interpreted as exploratory operating-region samples from the evaluated EMNIST setting, rather than as comprehensive deployment-level or generalized prosthetic-vision operating-envelope characterization, or as evidence of cross-benchmark stochastic robustness.

**Figure 10:**
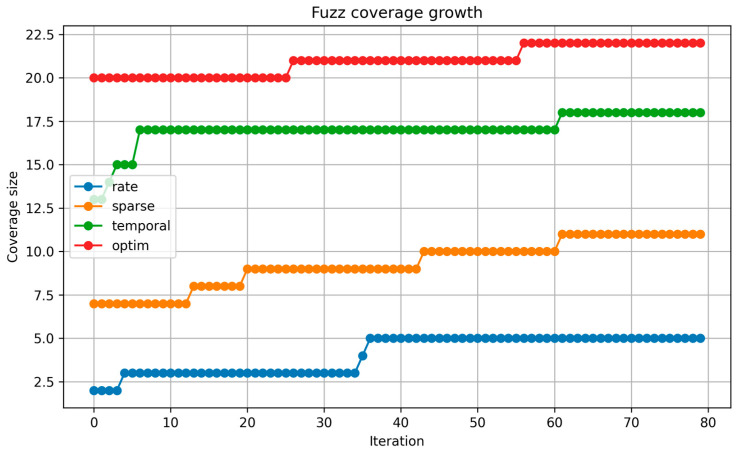
Fuzzing coverage growth on EMNIST Letters. Coverage-bin accumulation across stochastic single-operator mutations for the symbolic-recognition benchmark. These results represent exploratory benchmark-specific operating-region sampling rather than deployment-level operating-envelope characterization.

Coverage-growth curves are shown in Figure 10. The fuzzing results indicate that coverage expanded rapidly at the beginning of the search and then saturated early, with substantial encoder-dependent differences in the final number of bins discovered. The optim encoder achieved the largest final coverage size (22), followed by temporal (18), sparse (11), and rate (5). For temporal and optim, most of the reachable coverage was already discovered within the first few iterations, after which only occasional new bins were added. The rate encoder saturated most strongly, reaching a final coverage size of only 5 and showing no new coverage after iteration 36. Sparse and temporal continued to add a small number of new bins until iteration 61, but the overall growth remained modest after the early phase. Thus, within the fixed mutation budget used in the present EMNIST run, coverage growth stabilized after the early iterations and yielded a relatively compact set of sampled stress-sensitive coverage bins.

**Figure 11:**
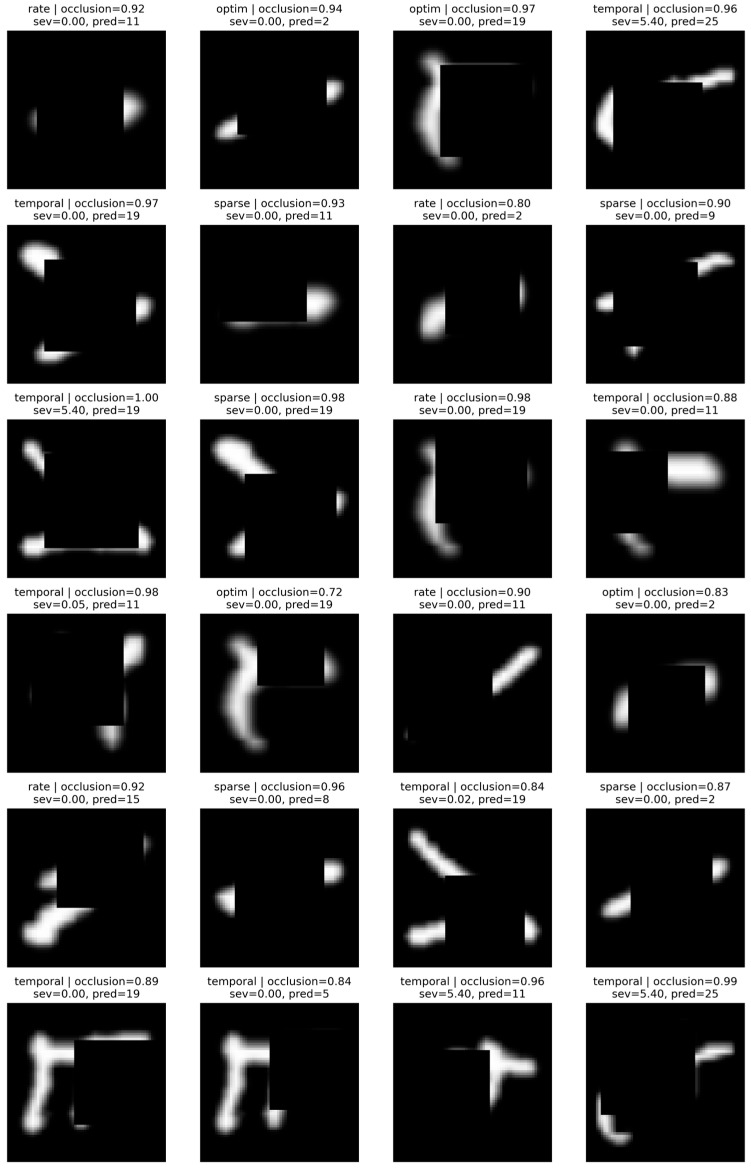
Top-ranked fuzzing counterexamples on EMNIST Letters. Representative severely degraded mutated cases for the symbolic-recognition benchmark.

The top-ranked counterexamples were concentrated in one perturbation family. All 24 entries in Supplementary Table S7 corresponded to occlusion, with stress levels between approximately 0.72 and 1.00. This concentration was consistent with the structured-sweep pattern in Section 5.1, where severe occlusion also produced the largest observed performance degradation. Within the implemented EMNIST single-operator fuzzing configuration, however, these cases should be interpreted only as candidate stress-sensitive samples near the upper end of the occlusion range, rather than as an exhaustive characterization of the stochastic stress space. The counterexample scores were driven mainly by misclassification and representation displacement for rate, sparse, and optim. Several temporal cases additionally exhibited the maximum observed residual proxy severity of 5.4. Thus, the sampled cases illustrate different contributions of task failure, representation displacement, and residual proxy burden within this limited benchmark-specific configuration. They should not be generalized to multi-operator perturbation interactions or deployment-level prosthetic-vision operating conditions. At the same time, the counterexample table reveals that high representation displacement can remain a major component of the composite counterexample score even when residual proxy safety burden is negligible, reinforcing the need to keep topology-based representation variation in the evaluation loop. Figure 11 shows representative top-ranked counterexamples generated by the fuzzing process on the EMNIST Letters benchmark, illustrating stress-sensitive cases that lead to severe degradation under structured stress.

Accordingly, the current fuzzing results complement the structured sweep by highlighting a candidate degradation-sensitive operating region and by showing how task failure, representation displacement, and residual proxy burden contributed to the sampled counterexample scores.

The predominance of occlusion-driven counterexamples in the stochastic fuzzing analysis should not be interpreted as surprising, since contiguous information removal is generally expected to produce stronger degradation than distributed perturbations such as blur or additive noise. However, the present results suggest that phosphene-constrained representations may be particularly sensitive to large contiguous percept loss because local information removal cannot be compensated through dense spatial redundancy to the same extent as in conventional high-resolution image representations. Consequently, occlusion perturbations tended to generate disproportionately severe degradation regions in both the functional and representation-level analyses.

Because COCO-derived fuzzing artifacts were not generated in the present run, the conclusions of this section should be interpreted as benchmark-specific rather than cross-benchmark. The present results therefore represent exploratory operating-region sampling within the evaluated EMNIST single-operator fuzzing scope and implemented stress configuration, rather than comprehensive characterization of all possible SPV perturbation interactions, generalized prosthetic-vision stochastic stress behavior, or deployment-level operating envelopes. Nevertheless, within this limited scope, the EMNIST results suggest that stochastic single-operator exploration can provide useful complementary information beyond the structured grid sweep, especially for highlighting candidate stress-sensitive regions and representative counterexamples for qualitative inspection.

### 5.7. Summary of Findings

The main empirical findings can be summarized along five axes.

First, structured stress sweeps revealed benchmark-dependent degradation trajectories that were not recoverable from clean-condition accuracy alone. In EMNIST, stress induced a broad and clearly separated degradation range, especially under noise, dropout, and occlusion, whereas the COCO-derived benchmark operated in a lower but more compressed accuracy regime in which encoder differences were less pronounced at the level of performance alone.

Second, residual proxy safety burden after feasibility projection did not track performance monotonically, making it necessary to report performance and residual proxy safety burden jointly rather than sequentially. The relationship between performance and residual proxy safety burden differed across benchmarks, with weaker alignment observed under the compressed low-accuracy conditions of the COCO-derived benchmark. In particular, sparse consistently maintained an almost negligible residual proxy safety burden, whereas temporal incurred the largest residual proxy safety-burden cost and rate and optim occupied an intermediate regime.

Third, topology-based representation metric provided a complementary structural diagnostic and, in the COCO-derived benchmark, revealed reduced coupling between topology-based representation metric and decoder accuracy under degraded COCO-derived conditions where output-level accuracy variations remained comparatively compressed. In EMNIST, topology-based representation metric largely tracked the degradation of task performance and therefore acted mainly as a structurally informative companion metric. In contrast, the COCO-derived benchmark showed a substantially larger variation in topology-based representation metrics than in output-level accuracy under degraded conditions.

Fourth, the tri-objective summaries showed that operating-point selection was more informative than single-metric ranking. Under the baseline equal-weight policy, the sparse encoder achieved the highest point-estimate tri-objective utility across both benchmarks, but the bootstrap analysis indicates that this observation should not be interpreted as statistically conclusive in EMNIST. The additional Pareto-front membership analysis further showed that the encoders should be interpreted as occupying different trade-off regions rather than forming a single absolute hierarchy. In particular, sparse occupied a favorable proxy-feasibility-controlled region because of its near-zero residual proxy safety burden, whereas rate, temporal, and optim retained competitive stress-integrated performance or topology-based representation metrics in benchmark-specific regimes.

Fifth, stochastic fuzzing provided exploratory sampling beyond the structured grid sweep by highlighting candidate stress-sensitive regions and representative counterexamples in the EMNIST benchmark. Within the evaluated single-operator configuration, the top-ranked sampled cases were concentrated under severe occlusion, which was consistent with the adverse occlusion pattern observed in the structured sweep. Their counterexample scores were driven primarily by task failure and representation displacement, while the temporal encoder also showed elevated residual proxy burden in some sampled cases. Because benchmark-specific fuzzing artifacts were generated only for EMNIST and under the present single-operator sampling design, these observations should be interpreted as exploratory and benchmark-specific rather than as cross-benchmark, multi-operator, or deployment-level characterization. Taken together, these results are consistent with the more limited methodological claim that nominal-condition accuracy or any single output metric is insufficient for fully characterizing encoder behavior under the evaluated stress conditions. Across the present experiments, sparse emerged as a favorable point-estimate configuration under the baseline equal-weight utility and fixed-decoder evaluation setting, primarily because it combined competitive performance and topology-based representation metrics with an exceptionally low residual proxy safety burden. More broadly, the results suggest that prosthetic-vision encoder assessment may benefit from simultaneous analysis of output-level performance, proxy feasibility-constrained behavior, and topology-based representation variation under structured perturbation, rather than from relying on a single performance metric alone.

The reduced COCO-derived benchmark should be interpreted cautiously because decoder accuracy remained relatively close to the four-class chance baseline under several degraded conditions. Accordingly, the observed dissociation trends should be interpreted only within the reduced four-class COCO-derived benchmark and the evaluated simulation setting.

## 6. Conclusions

This study presents a stress-based evaluation framework that reframes phosphene encoder assessment as a tri-objective operating-envelope problem. Instead of evaluating phosphene encoders only at nominal conditions, the proposed framework combined structured stress sweeps, residual proxy safety-burden analysis, topology-based representation metrics, and stress-integrated utility summaries to characterize how different encoders behave under progressively adverse conditions. In this sense, the main contribution of the present work is methodological: it provides a practical and reproducible way to compare phosphene encoders in terms of stressed functional performance, proxy feasibility-constrained behavior, and topology-based representation metrics within a unified simulation analysis pipeline.

The empirical results are consistent with the central methodological premise of the study. First, clean-conditioning accuracy alone was not sufficient to predict encoder behavior under stress. The EMNIST benchmark showed a broad degradation range with substantial operator-dependent separation, whereas the COCO-derived benchmark operated in a lower but more compressed performance regime. Second, performance and residual proxy safety burden were only loosely aligned. Across both benchmarks the sparse encoder yielded the most favorable point-estimate utility values under the baseline equal-weight evaluation policy; however, because the benchmark-specific decoder was trained on clean-condition percepts and kept fixed across stress conditions, this observation should be interpreted with both the uncertainty qualifications reported in the results section and the fixed-observer limitation of the present evaluation design. Third, the exploratory topology-based representation analysis provided complementary representation-level diagnostic information beyond output-level metrics. In EMNIST, the exploratory topology-based representation metric largely tracked performance degradation and therefore acted as a structurally informative companion diagnostic. In the COCO-derived benchmark, however, topology-based representation metrics showed larger relative variation than decoder accuracy within the evaluated simulation setting under degraded conditions. These results should be interpreted as exploratory topology-based diagnostics rather than as validation of a general representation-stability measure. Overall, these results indicate that encoder performance, residual proxy safety burden, and topology-based representation metrics were only partially aligned within the evaluated simulation setting, suggesting that they should be considered jointly in stress-based simulation analyses.

Taken together, these findings indicate that phosphene encoder evaluation should be treated as a multi-axis decision problem. An encoder that appears acceptable under nominal or accuracy-only evaluation may nevertheless accumulate comparatively large residual proxy safety burden or exhibit topology-based representation variation under stress conditions that are not fully reflected in output-level decoder metrics within the present simulation setting. From this perspective, the value of the proposed framework lies not only in ranking encoders, but in exposing how that ranking depends on the balance among stressed functional performance, residual proxy safety burden, and topology-based representation structure. The present experiments therefore suggest that encoder comparison under the evaluated simulation conditions may benefit from operating-envelope characterization rather than isolated benchmark scores alone.

Several limitations should be noted. First, the experiments were conducted in a simulated prosthetic vision setting with simplified phosphene generation and benchmark-specific shared decoders, rather than in hardware-in-the-loop or clinical stimulation environments. The observed encoder ranking and utility results are therefore specific to this simplified SPV simulation setting and cannot be generalized to real-world physiological or clinical prosthetic-vision conditions. Accordingly, the reported safety-related quantities should be interpreted as residual proxy safety-burden summaries after feasibility projection rather than as direct electrochemical, physiological, or clinical safety guarantees. The present framework should not be interpreted as a clinically calibrated neurostimulation safety model.

Second, the reduced COCO-derived four-class subset was sufficient for demonstrating benchmark dependence within the evaluated simulation setting, but it does not cover the diversity of real-world prosthetic-vision tasks, such as navigation, object localization, mobility support, reading fluency, scene segmentation, and obstacle avoidance. Third, the stochastic fuzzing analysis in the present run yielded benchmark-specific artifacts only for EMNIST and was based on single-operator sampling, so the fuzzing conclusions should be interpreted cautiously as exploratory operating-region samples rather than as comprehensive deployment-level operating-envelope characterization across tasks or perturbation combinations. Finally, the current framework remains offline and benchmark-centered; it does not yet incorporate user-specific calibration, adaptive stimulation policies, or closed-loop personalization. The present study also did not include broader robustness analysis across PCA dimensionality, TDA subset size, random initialization, persistence-distance variants, H0/H1 decomposition, or alternative representation-similarity frameworks such as CKA and RSA. Accordingly, the present topology-based analyses should be interpreted as exploratory representation-level diagnostics within the evaluated simulation framework rather than as completed robustness validation of the topology pipeline.

The present study primarily focused on deterministic structured stress-sweep analysis and comparative operating-envelope characterization within a simplified simulation-based evaluation framework rather than statistical population inference across repeated randomized training procedures. Because the decoder remained fixed after clean-condition training, the present framework does not fully disentangle encoder-specific degradation from downstream decoder sensitivity to stressed percept distributions. Accordingly, the reported encoder rankings and utility comparisons should be interpreted as uncertainty-qualified, benchmark-specific, and configuration-dependent joint encoder–decoder observations within the evaluated fixed-decoder simulation setting, rather than as universally generalizable or encoder-only robustness claims. Although we added Wilson confidence intervals for accuracy and condition-level bootstrap confidence intervals for utility based on the current single-run outputs, the present study still did not include repeated-seed uncertainty estimation, repeated decoder training, bootstrap-supported latent-fragility threshold calibration, effect-size analysis, or condition-level significance testing. Therefore, the reported encoder rankings should be interpreted as uncertainty-qualified, configuration-dependent observations rather than statistically definitive comparative conclusions.

These limitations point directly to future work. A first priority is to extend the framework to richer task suites and more realistic phosphene simulators, including settings in which calibration drift, electrode dropout, or subject-specific phosphene maps can be modeled explicitly. Future work should extend the proposed stress-analysis framework to richer prosthetic-vision task suites, including navigation-oriented, localization-aware, mobility-support, reading-fluency, scene-segmentation, and obstacle-avoidance benchmarks under more realistic SPV conditions. Future work should investigate whether the proposed stress-analysis framework and the observed encoder trade-off patterns remain stable under biologically informed phosphene simulators such as pulse2percept or differentiable phosphene-generation models, including subject-specific phosphene maps, implant-aware constraints, and temporal percept dynamics. Future work should also investigate robustness-oriented TDA validation, H0/H1 decomposition analysis, alternative persistence-distance formulations, and comparative evaluation against standard representation-similarity metrics such as CKA and RSA. Future work should investigate statistically calibrated fragility thresholds, uncertainty-aware latent-fragility analysis, and broader condition-level SPV stress characterization. Future work should also extend stochastic fuzzing to the COCO-derived benchmark and to multi-operator perturbation combinations in order to evaluate broader operating-region coverage beyond the current EMNIST-only single-operator sampling setting. Future work should investigate encoder-specific decoders, stress-augmented decoder adaptation, and representation-level linear-probe evaluation for disentangling encoder degradation from downstream decoder sensitivity. Future work should also incorporate repeated-seed robustness analysis, bootstrap-supported confidence intervals, effect-size and significance testing, confidence-aware utility-ranking criteria, and more exhaustive multi-objective decision analysis, including explicit Pareto-front estimation, dominance-depth analysis, and confidence-aware treatment of Pareto-front membership. A second priority is to integrate the present stress-based analysis with personalized and closed-loop operating policies so that encoder selection can adapt to individual perceptual structure and changing safety margins over time. A third priority is to investigate whether topology-based representation variation metrics, as captured here through topology-based metrics, can provide complementary topology-based representation diagnostics within realistic prosthetic-vision pipelines, including differentiable simulation environments and hardware-aware implementations. Ultimately, the present results suggest that phosphene encoder assessment may benefit from operating-envelope-oriented analysis in which stress-conditioned performance, residual proxy safety burden, and topology-based representation metrics are considered jointly rather than analyzed in isolation.

## Figures and Tables

**Table 1:** Benchmarks, compared encoding strategies, shared decoder configuration, and common evaluation settings. Summary of the two benchmark tasks, the four compared encoders, the benchmark-specific shared decoder, and the fixed preprocessing and operating conditions used throughout the experiments.

Category	Item	Specification
Benchmark suite	Benchmark A	EMNIST Letters
	Task	26-class symbolic recognition
	Final split	20,000/3000/3000 (train/validation/test)
	Benchmark B	COCO-derived balanced subset
	Task	4-class reduced COCO-derived image-level classification task (none, person, car, both)
	Final split	4000/1000/1000 (train/validation/test)
Reproducibility	Random seed	42
Preprocessing	Input format	Grayscale, normalized to [0,1], centered on a 64×64 canvas
	Electrode grid	16×16
	Percept size	32×32
	Percept synthesis	Nearest-neighbor upsampling followed by Gaussian smoothing
	Gaussian blur in percept synthesis	σ=1.0
Encoding strategy	rate	Rate-based amplitude mapping with image-statistics-driven global f and pw
	sparse	Sparse electrode selection with diversity constraint and shared global f–pw policy
	temporal	Latency modulation with exponential time-weighted integration
	optim	Iterative constrained optimization with soft-threshold sparsification and feasibility projection
Shared decoder	Scope	One shared decoder per benchmark, fixed across encoders and stress conditions
	Architecture	CNN(1→16→32) + FC(64) + Linear$\left(n_{classes}\right)$
	Input	32×32 percept image
	Optimizer	Adam
	Loss	Cross-entropy
	Epochs	8
	Batch size	128
	Learning rate	$10^{-3}$
Decoder training protocol	Training distribution	Clean percepts pooled across all four encoders within each benchmark
Operating bounds	Amplitude range	a∈[0,1]
	Frequency range	f∈[10,60] Hz
	Pulse-width range	pw∈[50,500] μs
Feasibility-projection constraints	Duty-cycle limit	$duty_{max}=0.10$
	Proxy charge limit	$q_{max}=0.015$
	Sparsity limit	$k_{max}=40$ active electrodes
Stress protocol	Non-clean operators	noise, blur, dropout, occlusion
	Stress levels	0.00,0.25,0.50,0.75,1.00

**Table 2:** COCO-derived clean-condition overall diagnostic metrics and chance-baseline comparison. Values were computed from the final fixed-seed canonical dataset. The four-class random-chance baseline is 0.25.

Encoder	Accuracy	Balanced Accuracy	Macro-F1	Four-Class Chance Baseline	Accuracy-Chance
**rate**	0.3010	0.3010	0.2932	0.2500	0.0510
**sparse**	0.3000	0.3000	0.2892	0.2500	0.0500
**temporal**	0.3040	0.3040	0.2970	0.2500	0.0540
**optim**	0.3000	0.3000	0.2920	0.2500	0.0500

**Table 3:** Per-class diagnostic classification metrics for the reduced four-class COCO-derived benchmark under the clean condition.

Encoder	Class	Precision	Recall	F1-Score	Support
**rate**	none	0.2756	0.3120	0.2927	250
	person	0.3109	0.2960	0.3033	250
	car	0.3186	0.4320	0.3667	250
	both	0.2929	0.1640	0.2103	250
**sparse**	none	0.2594	0.4160	0.3195	250
	person	0.3263	0.3720	0.3477	250
	car	0.3167	0.2800	0.2972	250
	both	0.3548	0.1320	0.1924	250
**temporal**	none	0.2818	0.3280	0.3031	250
	person	0.3182	0.2800	0.2979	250
	car	0.3224	0.4320	0.3692	250
	both	0.2857	0.1760	0.2178	250
**optim**	none	0.2766	0.3120	0.2932	250
	person	0.3064	0.2880	0.2969	250
	car	0.3178	0.4360	0.3676	250
	both	0.2929	0.1640	0.2103	250

**Table 4:** Clean-condition confusion matrices for the reduced four-class COCO-derived benchmark. Rows correspond to true labels, and columns correspond to predicted labels. Class order: none, person, car, both.

Encoder	True Class	Pred. None	Pred. Person	Pred. Car	Pred. Both
rate	none	78	69	74	29
	person	76	74	64	36
	car	65	43	108	34
	both	64	52	93	41
sparse	none	104	80	52	14
	person	105	93	35	17
	car	103	48	70	29
	both	89	64	64	33
temporal	none	82	64	74	30
	person	76	70	62	42
	car	65	39	108	38
	both	68	47	91	44
optim	none	78	69	75	28
	person	75	72	66	37
	car	64	43	109	34
	both	65	51	93	41

**Table 5:** Clean-condition performance and residual proxy safety-burden summaries across the two benchmarks. Encoder-wise clean-condition accuracy and residual proxy safety-burden summaries for EMNIST and the COCO-derived benchmark.

Benchmark	Encoder	Accuracy	Max Sev.	Mean Sev.	Viol. Rate
EMNIST	rate	0.8000	0.2000	0.0010	0.4387
	sparse	0.7730	2.26 × 10^−7^	2.65 × 10^−8^	0.4120
	temporal	0.7933	5.4000	0.0172	0.4287
	optim	0.8020	0.2000	0.0009	0.4303
COCO	rate	0.3010	0.5000	0.0038	0.4870
	sparse	0.3000	2.26 × 10^−7^	2.73 × 10^−8^	0.4140
	temporal	0.3040	0.5000	0.0038	0.4000
	optim	0.3000	0.5000	0.0038	0.4590

**Table 6:** Worst-case stress-sweep performance and residual proxy safety burden across the two benchmarks. Encoder-wise minima and maxima derived over the full structured stress grid for EMNIST and the COCO-derived benchmark.

Benchmark	Encoder	Worst Acc.	Acc. Op	Level	Max Sev.	Sev. Op	Level
EMNIST	rate	0.1383	occlusion	1.00	0.2250	blur	0.50
	sparse	0.1433	occlusion	1.00	2.88 × 10^−7^	blur	0.50
	temporal	0.1297	occlusion	1.00	5.4000	clean	0.00
	optim	0.1437	occlusion	1.00	0.2250	blur	0.50
COCO	rate	0.2890	occlusion	1.00	0.5000	clean	0.00
	sparse	0.2670	noise	0.75	2.88 × 10^−7^	blur	0.25
	temporal	0.2780	noise	1.00	5.4000	occlusion	1.00
	optim	0.2590	dropout	1.00	0.5000	clean	0.00

**Table 7:** Coupling statistics across the two benchmarks. Encoder-wise correlation summaries among accuracy, residual severity, and the topology-based representation metric for EMNIST and the COCO-derived benchmark.

Benchmark	Encoder	Corr(TSI, Acc)	Corr(TSI, Sev)	Corr(Acc, Sev)
EMNIST	rate	0.8069	0.5127	0.1982
	sparse	0.8002	0.0784	0.1807
	temporal	0.8421	0.0778	−0.17
	optim	0.8198	0.692	0.257
COCO	rate	0.6776	0.6453	0.1851
	sparse	0.6022	0.129	0.0903
	temporal	−0.0356	0.3732	−0.366
	optim	0.259	0.6658	0.0832

**Table 8:** Tri-objective summary across the two benchmarks. Benchmark-tagged encoder-wise stress-integrated and worst-case summaries for EMNIST and the COCO-derived benchmark, regenerated from the final fixed-seed canonical dataset.

Benchmark	Encoder	Min. Acc.	Max. Sev.	Min. TSI	TSI_P	TSI_R	R_Safe	Utility
EMNIST	rate	0.1383	0.2250	0.0060	0.8318	0.6427	0.1609	1.3136
EMNIST	sparse	0.1433	2.88 × 10^−7^	0.0722	0.7653	0.6495	2.86 × 10^−7^	1.4148
EMNIST	temporal	0.1297	5.4000	0.0782	0.8178	0.6160	4.0906	−2.6568
EMNIST	optim	0.1437	0.2250	0.0158	0.8342	0.6039	0.1438	1.2944
COCO	rate	0.2890	0.5000	0.0385	0.3780	0.5674	0.3156	0.6298
COCO	sparse	0.2670	2.88 × 10^−7^	0.1498	0.3742	0.6486	2.86 × 10^−7^	1.0228
COCO	temporal	0.2780	5.4000	0.0433	0.3798	0.6001	0.6016	0.3784
COCO	optim	0.2590	0.5000	0.0206	0.3721	0.5436	0.3125	0.6032

**Table 9:** Bootstrap confidence intervals for tri-objective utility under the baseline equal-weight evaluation policy. Point-estimate utilities correspond to the deterministic stress-integrated summaries reported in Table 8, whereas bootstrap means and confidence intervals were computed using condition-level percentile bootstrap resampling over the non-clean encoder-level and operator-level rows.

Benchmark	Encoder	Point-Estimate Utility	Bootstrap Mean Utility	95% CI Lower	95% CI Upper	n Bootstrap
EMNIST	rate	1.3136	1.3136	1.0366	1.5263	5000
	sparse	1.4148	1.4148	1.1112	1.6240	5000
	temporal	−2.6568	−2.6568	−4.1616	−1.0803	5000
	optim	1.2944	1.2944	1.0231	1.4940	5000
COCO	rate	0.6298	0.6298	0.4549	0.7686	5000
	sparse	1.0228	1.0228	0.8338	1.1278	5000
	temporal	0.3784	0.3784	−0.5489	0.7731	5000
	optim	0.6032	0.6032	0.4530	0.7322	5000

**Table 10:** Utility sensitivity analysis under alternative operating-preference weights. Post hoc utility values computed under balanced, performance-dominant, topology-metric-dominant, and proxy-burden-dominant weighting scenarios using P = TSI_P, R = TSI_R, and S = 1 − R_safe. The table evaluates whether encoder ranking changes when the operating preference shifts toward performance, topology-based representation metrics, or residual proxy safety-burden control.

Benchmark	Encoder	Preference Scenario			
		Balanced	Performance	Topology Metric	Proxy Burden
EMNIST	rate	0.7712	0.7955	0.7198	0.7983
	sparse	0.8049	0.7891	0.7428	0.8830
	temporal	−0.5523	−0.0043	−0.0850	−1.5676
	optim	0.7648	0.7926	0.7005	0.8014
COCO	rate	0.5433	0.4772	0.5529	0.5997
	sparse	0.6743	0.5543	0.6640	0.8046
	temporal	0.4595	0.4276	0.5157	0.4351
	optim	0.5344	0.4695	0.5381	0.5956

**Table 11:** Pareto-style dominance summary across the three stress-integrated objective axes. TSI_P and TSI_R were treated as maximization objectives, whereas R_safe was treated as a minimization objective. An encoder was considered dominated if another encoder achieved equal or better values on all three axes and a strictly better value on at least one axis.

Benchmark	Encoder	TSI_P	TSI_R	R_Safe	Pareto-Front Membership	Dominance Status	Dominated by
EMNIST	rate	0.8318	0.6427	0.1609	Yes	non-dominated	none
EMNIST	sparse	0.7653	0.6495	0.0000	Yes	non-dominated	none
EMNIST	temporal	0.8178	0.6160	4.0906	No	dominated	rate
EMNIST	optim	0.8342	0.6039	0.1438	Yes	non-dominated	none
COCO	rate	0.3780	0.5674	0.3156	Yes	non-dominated	none
COCO	sparse	0.3742	0.6486	0.0000	Yes	non-dominated	none
COCO	temporal	0.3798	0.6001	0.6016	Yes	non-dominated	none
COCO	optim	0.3721	0.5436	0.3125	No	dominated	sparse

## Data Availability

EMNIST Letters and MS COCO are publicly available datasets. The processed result artifacts, exported tables, figures, Supplementary Materials, and analysis notebook used in this study are available in the public GitHub repository listed in the Supplementary Materials section.

## Supplementary Materials

The fixed-seed reproducibility package for the EMNIST Letters and reduced four-class COCO-derived benchmarks, including benchmark configurations, saved split metadata, trained decoder files, condition-level result tables, figures, bootstrap confidence-interval outputs, Pareto-front and dominance analyses, weight-sensitivity outputs, run manifests, and the canonical executable notebook, is available at the GitHub repository: [https://github.com/yslee9864/Stress-Based-Validation-of-Bio-Inspired-Phosphene-Vision-Encoding] (accessed on 27 June 2026).

## Abbreviations

The following abbreviations are used in this manuscript:

CKA	Centered Kernel Alignment
CNN	Convolutional Neural Network
COCO	Common Objects in Context
CVaR	Conditional Value-at-Risk
EMNIST	Extended Modified National Institute of Standards and Technology
FC	Fully Connected
PCA	Principal Component Analysis
RSA	Representational Similarity Analysis
SPV	Simulated Prosthetic Vision
TSI	Topology-based representation diagnostic index; retained as an implementation label for the normalized topology-based representation metric
VCP	Visual Cortical Prosthesis
